# DNA bending facilitates the error-free DNA damage tolerance pathway and upholds genome integrity

**DOI:** 10.1002/embj.201387425

**Published:** 2014-01-31

**Authors:** Victor Gonzalez-Huici, Barnabas Szakal, Madhusoodanan Urulangodi, Ivan Psakhye, Federica Castellucci, Demis Menolfi, Eerappa Rajakumara, Marco Fumasoni, Rodrigo Bermejo, Stefan Jentsch, Dana Branzei

**Affiliations:** 1 Fondazione Istituto FIRC di Oncologia Molecolare (IFOM)Milan, Italy; 2 Department of Molecular Cell Biology, Max Planck Institute of BiochemistryMartinsried/Munich, Germany

**Keywords:** chromatin architecture, DNA damage tolerance, mutagenesis, replication, template switching

## Abstract

DNA replication is sensitive to damage in the template. To bypass lesions and complete replication, cells activate recombination-mediated (error-free) and translesion synthesis-mediated (error-prone) DNA damage tolerance pathways. Crucial for error-free DNA damage tolerance is template switching, which depends on the formation and resolution of damage-bypass intermediates consisting of sister chromatid junctions. Here we show that a chromatin architectural pathway involving the high mobility group box protein Hmo1 channels replication-associated lesions into the error-free DNA damage tolerance pathway mediated by Rad5 and PCNA polyubiquitylation, while preventing mutagenic bypass and toxic recombination. In the process of template switching, Hmo1 also promotes sister chromatid junction formation predominantly during replication. Its C-terminal tail, implicated in chromatin bending, facilitates the formation of catenations/hemicatenations and mediates the roles of Hmo1 in DNA damage tolerance pathway choice and sister chromatid junction formation. Together, the results suggest that replication-associated topological changes involving the molecular DNA bender, Hmo1, set the stage for dedicated repair reactions that limit errors during replication and impact on genome stability.

## Introduction

Damaged DNA templates are major obstacles during replication, inducing fork stalling and discontinuities in the replicated chromosomes. DNA damage tolerance (DDT) mechanisms are crucial to promote replication completion by mediating fork restart and filling of DNA gaps (Lopes *et al*, 2006; Branzei *et al*, 2008; Daigaku *et al*, 2010; Karras & Jentsch, 2010; Minca & Kowalski, 2010). Genetic work has delineated two main modes of DDT in all organisms: an error-free mode involving recombination in which one newly synthesized strand is used as a template for replication of the blocked nascent strand, and an error-prone mode involving translesion synthesis (TLS) and which is largely accountable for mutagenesis (reviewed in Friedberg, 2005; Branzei, 2011). Because increased mutations ultimately lead to genome instability and cancer (Nik-Zainal *et al*, 2012; Alexandrov *et al*, 2013), the molecular mechanisms underlying DDT pathway choice have implications for understanding cancer etiology and for cancer therapy. At present, the mechanisms underlying the error-free/error-prone DDT pathway switch remain little understood: on one hand, high expression of TLS polymerases in mitosis may represent a passive mechanism that favors error-free damage-bypass early during replication (Waters & Walker, 2006), in line with the observed correlation between replication timing and mutation rates (Lang & Murray, 2011); on the other hand, regulatory mechanisms, such as the ones involving post-translational modification of the polymerase clamp, PCNA, with SUMO and ubiquitin, modulate the recruitment of repair factors and TLS polymerases, thus influencing DDT pathway choice (Bergink & Jentsch, 2009).

PCNA modifications with SUMO and ubiquitin are crucial for DDT: mono-ubiquitylation of PCNA promotes translesion polymerase-mediated error-prone DDT (Stelter & Ulrich, 2003), Rad5-Mms2-Ubc13-dependent polyubiquitylation of PCNA acts in conjunction with a subset of homologous recombination factors to mediate error-free DDT by formation of sister chromatid junctions (SCJs) (Branzei *et al*, 2008; Minca & Kowalski, 2010; Vanoli *et al*, 2010; Karras *et al*, 2013), and SUMOylated PCNA recruits Srs2 to chromatin, where it presumably prevents the access of the recombination machinery and inhibits unwanted recombination (Papouli *et al*, 2005; Pfander *et al*, 2005; Branzei *et al*, 2008; Karras *et al*, 2013). The recombination pathway prevented by SUMOylated PCNA is also known as the salvage pathway of DDT, whereas the Rad5-mediated pathway is commonly referred to as template switching. Notably, both these error-free DDT pathways mediate damage-bypass via the formation of SCJs, but may occupy distinct time windows in relation to DNA replication (Branzei *et al*, 2008; Karras *et al*, 2013).

Following the formation of damage-bypass SCJs, the Sgs1 helicase, homolog of human BLM that is mutated in cancer-prone Bloom syndrome patients, is thought to process together with the Top3 topoisomerase these intermediates to hemicatenanes, topological structures conjoining two DNA duplexes through a single-strand interlock, (Wu & Hickson, 2003; Liberi *et al*, 2005; Branzei *et al*, 2008; Karras & Jentsch, 2010; Cejka *et al*, 2012). Type IA topoisomerases—Top1 and Top3 in budding yeast—that catalyze strand passage through a reversible, enzyme-bridged, single-strand break can then resolve the resulting hemicatenanes (Wang, 2002). When Sgs1 functionality is impaired, the SCJs arising during error-free DDT are resolved by crossover-prone nucleases (Ashton *et al*, 2011; Szakal & Branzei, 2013), leading to elevated sister chromatid exchanges and loss of heterozygosity events that may ultimately drive chromosomal instabilities underpinning tumorigenesis (Wechsler *et al*, 2011; Szakal & Branzei, 2013).

High mobility group box (HMGB) proteins are abundant, multifunctional proteins with genome architectural capacity conferred by their ability to bend DNA, in the process creating DNA topologies that can impinge on the assembly of nucleoprotein structures (reviewed in Thomas & Travers, 2001; Stros, 2010). Notably, HMGB1 binds with high affinity to hemicatenanes (Stros *et al*, 2004; Jaouen *et al*, 2005). The *Saccharomyces cerevisiae* HMGB protein, Hmo1 - the closest ortholog of HMGB1 in yeast-, shows synthetic lethal interactions with *top3*Δ (Gadal *et al*, 2002), and binds with preference to single stranded (ss) DNA and to DNA with altered conformations, showing reduced DNA sequence specificity (Kamau *et al*, 2004; Bauerle *et al*, 2006; Xiao *et al*, 2010). In addition, in *hmo1* mutant cells, spontaneous and damage-induced mutagenesis is increased (Alekseev *et al*, 2002; Kim & Livingston, 2006, 2009), suggesting a possible role for Hmo1 in DDT or its regulation. It is of note that while mutation rates vary along chromosomes and correlate with replication timing (Lang & Murray, 2011), the underlying mechanisms accounting for the preferred usage of error-free DDT early in S phase remain elusive.

Here we show that Hmo1 has an early regulatory role, coincident with DNA replication, in error-free DDT pathway choice by channeling lesions towards the Rad5-Mms2-Ubc13-mediated pathway of template switching, while preventing mutagenic bypass and toxic recombination. We uncover that error-free DDT pathway choice, previously shown to be controlled by SUMOylated PCNA and its interactors Srs2 and Elg1, is uncoupled from the SCJ formation process *per se*. While Srs2 and Elg1 do not play a discernible role in SCJ formation, Hmo1 affects also this latter process. The time window for Hmo1 action in SCJ formation overlaps with the one of the Rad5-Mms2-Ubc13, being predominant early during replication. Importantly, these Hmo1 functions in error-free DDT are largely mediated via its carboxy (C)-terminal domain, previously shown to promote DNA bending. We additionally find that Hmo1 promotes topological transitions related to catenane/hemicatenane formation/stabilization during unperturbed growth and that this function is also largely dependent on its C-terminal domain. Together, the results indicate that the Hmo1-mediated topological pathway involving DNA bending represents a new replication-associated regulatory mechanism that facilitates error-free DDT and influences the error-free/error-prone DDT switch.

## Results

### Hmo1 functionally interacts with the Rad5-Mms2-Ubc13 error-free DDT pathway

Hmo1 and its human ortholog, HMGB1, exhibit high affinity for DNA hemicatenanes and other types of DNA with altered conformations such as ssDNA and DNA cruciform structures (Bianchi *et al*, 1989; Lu *et al*, 1996; Kamau *et al*, 2004; Jaouen *et al*, 2005) forming during replication in unperturbed and genotoxic stress conditions (Lopes *et al*, 2003, 2006; Liberi *et al*, 2005; Branzei *et al*, 2008). Hmo1 is an abundant protein, associated with chromatin throughout the cell-cycle (Bermejo *et al*, 2009). Following replication in the presence of DNA damage (MMS), we found by ChIP-on-chip a statistically significant co-localization between Hmo1 clusters and the ones of Rfa1, the large subunit of RPA (*P*-value 1.80E-16), which presumably marks ssDNA regions (Supplementary Fig S1A). Indeed, after treatment with high doses of HU, which blocks replication by depleting dNTP pools, Rfa1 peaks were clustered around early origins of replication and were overlapping with the BrdU peaks marking ongoing DNA replication (Supplementary Fig S1B, *P*-value 3.10E-17), in line with findings showing that HU treatment induces replication fork stalling and accumulation of ssDNA regions in the proximity of origins of replication (Sogo *et al*, 2002; Feng *et al*, 2006). On the other hand, following treatment with sublethal doses of MMS, which does not slow down replication fork progression to the same degree as high HU concentrations, Rfa1 peaks were spread over much larger regions (Supplementary Fig S1A), supporting the notion that during replication in the presence of genotoxic stress, DNA gaps persist behind replication forks (Lopes *et al*, 2006). Coating of ssDNA gaps with RPA facilitates the recruitment of the Rad18 ubiquitin ligase (Davies *et al*, 2008), which together with the Rad6 ubiquitin conjugating enzyme and the Rad5-Mms2-Ubc13 ubiquitylation complex, induces PCNA mono- and polyubiquitylation (Hoege *et al*, 2002) and mediates postreplicative DDT (Daigaku *et al*, 2010; Karras & Jentsch, 2010). The overlap between Hmo1 and Rfa1 clusters in MMS-treated cells (Supplementary Fig S1A), together with previous reports indicating a role for Hmo1 in the control of mutagenesis (Alekseev *et al*, 2002; Kim & Livingston, 2006), prompted us to investigate a possible involvement of Hmo1 in DDT and the metabolism of DNA structures arising during recombination-mediated damage-bypass.

**Figure 1:**
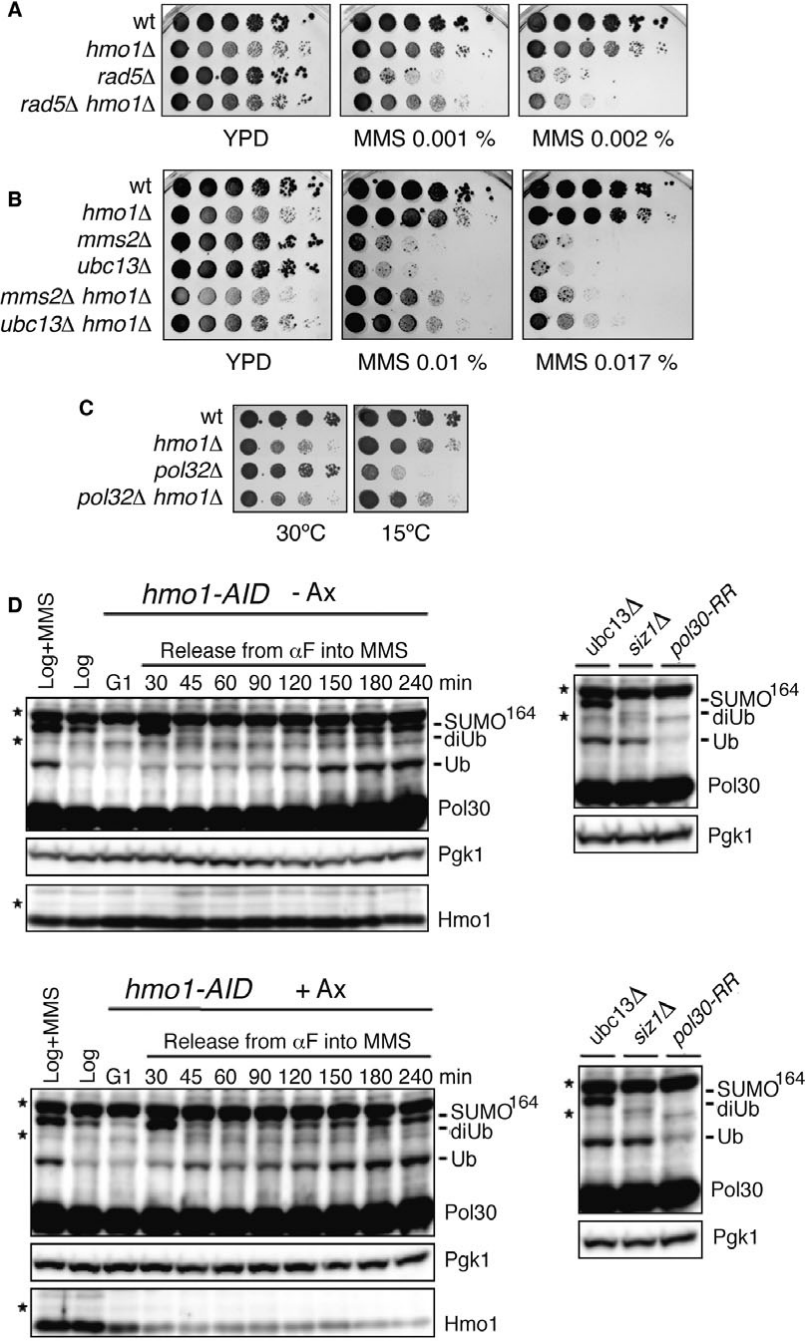
Hmo1 interacts functionally with the Rad5-Mms2-Ubc13 error-free DDT pathway

Two genetic pathways, the Rad51 and the Rad5-Mms2-Ubc13 pathways were identified to contribute to error-free DDT (Branzei *et al*, 2008; Karras *et al*, 2013). While *hmo1Δ* cells had wild-type (WT) levels of MMS resistance and the *hmo1Δ* mutation did not increase or rescue the MMS sensitivity of *rad51*Δ cells (data not shown and see below), it partially but discernibly suppressed the damage sensitivity of *rad5Δ* cells in two different yeast backgrounds, DF5 (Fig1A) and W303 (see below), suggesting a functional interaction between Hmo1 and Rad5. We further examined if this genetic relationship extended to other factors involved in PCNA polyubiquitylation. We found that the *hmo1Δ* mutation also partly suppressed the MMS sensitivity associated with null mutations in *MMS2* and *UBC13* (Fig1B), indicating that Hmo1 affects the usage of the Rad5-Mms2-Ubc13 error-free DDT pathway.

*HMO1* deletion rescues the MMS sensitivity of *rad5Δ*. wt (FY0113), *hmo1Δ* (HY3956), *rad5Δ* (HY0516), *rad5Δ hmo1Δ* (HY1518) cells were spotted.

*HMO1* deletion rescues the MMS sensitivity of *mms2Δ* and *ubc13Δ*. wt (FY0113), *hmo1Δ* (HY1508)*, mms2Δ* (HY0518)*, ubc13Δ* (FY1490)*, mms2Δ hmo1Δ* (HY1519)*,* and *ubc13Δ hmo1Δ* (HY3959) were spotted.

*HMO1* deletion rescues the cold sensitivity of *pol32Δ*. wt (FY0090), *hmo1Δ* (HY2714), *pol32Δ* (HY2719) and *hmo1Δ pol32Δ* (HY2706) were spotted.

Hmo1 does not affect PCNA modifications with ubiquitin and SUMO. Western blot of Pol30 (PCNA) in an *hmo1-AID* conditional mutant (HY2174) following or not Hmo1 depletion by addition of auxin (Ax) before G1 arrest and release into MMS-containing media. Ubiquitylated and SUMOylated species are indicated. Hmo1 depletion control and Pgk1, used as loading control, are shown below. To the right, controls for lack of PCNA polyubiquitylation (*ubc13Δ*, Y2620), or SUMOylation (*siz1Δ*, Y1630), or both (*pol30-RR*, FY1487). Asterisks denote cross-reactive proteins.

To further test Hmo1 implication in error-free DDT, we used a recently elucidated genetic readout (Karras & Jentsch, 2010). Deletion of *POL32*, encoding a nonessential subunit of the replicative DNA polymerase δ (Polδ) that is required for DNA synthesis during template switching (Vanoli *et al*, 2010), generates replication stress accompanied by cold sensitivity and induction of error-free DDT – and therefore of PCNA polyubiquitylation (Karras & Jentsch, 2010; Karras *et al*, 2013). Because mutations affecting PCNA polyubiquitylation (*mms2Δ*, *ubc13Δ*, *rad5Δ*, and *pol30-K164R*) suppress the cold sensitivity of *pol32Δ* cells (Karras *et al*, 2013), suppressors of the *pol32Δ* cold sensitivity phenotype are potentially new components or regulators of the error-free DDT pathway. We found that *hmo1Δ* also partly suppressed the slow growth phenotype at low temperatures of *pol32Δ* cells (Fig1C), similarly to mutations in other components of the PCNA polyubiquitylation pathway, although to a smaller degree than those mutations (Supplementary Fig S1C). We note that *hmo1*Δ was reported to suppress the temperature sensitivity of other DNA Polδ mutants (Kim & Livingston, 2009), thus resembling also in this respect deletions of *RAD18, RAD5* and *MMS2-UBC13* (Giot *et al*, 1997; Branzei *et al*, 2002, 2004).

We then analyzed if Hmo1 affects PCNA post-translational modifications. Because *hmo1*Δ strains are slow growing, showing slower progression throughout the cell-cycle (Lu *et al*, 1996), and PCNA modifications with SUMO and ubiquitin are expected to be sensitive to cell-cycle changes and replication delays (Hoege *et al*, 2002), we established a conditional degron system (*hmo1-AID)*, in which Hmo1 depletion is induced by addition of auxin (Nishimura *et al*, 2009). Reduced levels of Hmo1 did not discernibly affect PCNA modifications with ubiquitin and SUMO (Fig1D), suggesting that the effects manifested by Hmo1 on the Rad5-mediated error-free DDT pathway (Fig1A and B) are not caused by alterations in PCNA modifications.

### Hmo1 roles in DDT regulation and SCJ formation are manifested during DNA replication

**Figure 2:**
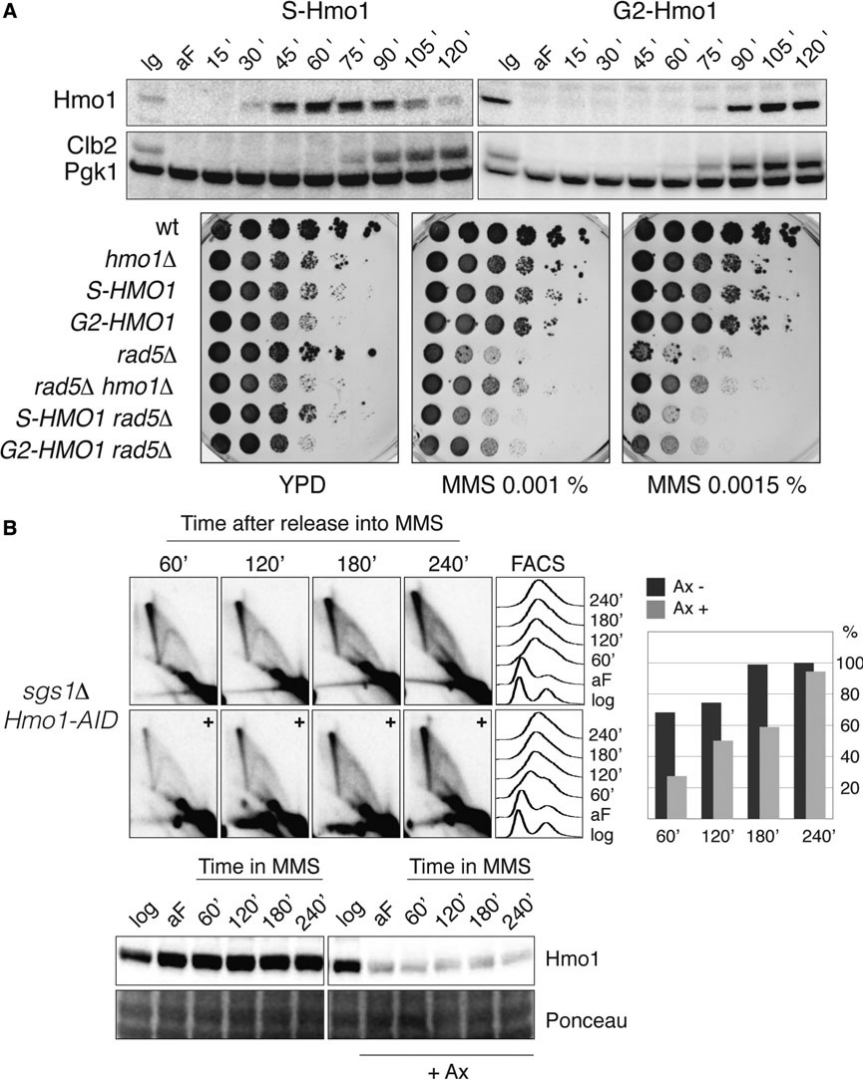
The roles of Hmo1 in Rad5 pathway regulation and SCJ formation are manifested during DNA replication

While the ability of cells to deal with exogenous DNA damage is not affected by restricting the expression of key DDT genes to the G2/M phase of the cell-cycle (Daigaku *et al*, 2010; Karras & Jentsch, 2010), other results suggest an early role for the Rad5 pathway during replication and SCJ formation (Branzei *et al*, 2008; Minca & Kowalski, 2010; Karras *et al*, 2013). To address if the role(s) of Hmo1 in regulating the Rad5 pathway (see Fig1) are normally manifested in S- or G2/M phases of the cell-cycle, or independently of the cell-cycle phase, we applied the S and G2 tags to *HMO1*. These tags restrict the expression of tagged proteins to specific phases of the cell-cycle, due to control elements of cyclin Clb6 or Clb2, respectively (Karras & Jentsch, 2010; Hombauer *et al*, 2011). When the S-tag- and G2-tag-containing DNA cassettes were integrated directly upstream of the *HMO1* open reading frame at its endogenous locus, the resulting fusion proteins were indeed largely restricted during the cell-cycle as assessed by comparing the expression of these proteins with the ones of Clb2 (Fig2A). When we further combined these *hmo1* alleles with a *rad5*Δ mutation, we found that specifically the *G2-HMO1* allele resembled *hmo1*Δ in its ability to suppress *rad5*Δ MMS sensitivity. Thus, Hmo1 role in regulating the Rad5 pathway is manifested during replication.

S-tag *HMO1* (*S-HMO1*, HY4324) and G2-tag *HMO1* (*G2-HMO1*, HY4325) cells were arrested in G1 phase and released into YPD at 28°C. Samples were collected at the indicated time points for Western blot analysis. The cell cycle progression was monitored using anti-Clb2 antibody; Pgk1 was used for loading control. Specifically the *G2-HMO1* allele partially rescues the MMS sensitivity of *rad5Δ* cells. wt (FY1296), *hmo1Δ* (HY1507), *S-HMO1* (HY4324), *G2*-*HMO1* (HY4325), *rad5Δ* (HY2682), *rad5Δ hmo1Δ* (HY3633), *S-HMO1 rad5Δ* (HY4355) and *G2*-*HMO1 rad5Δ* (HY4359) were spotted.

Hmo1 promotes SCJ formation during template switching in S phase. *HMO1-AID sgs1Δ* (HY2176) cells were synchronized with alpha-factor (aF) and divided into two identical parts. One half of the culture was treated with auxin and released into YPD media containing 0.033% MMS in the presence of auxin (+), the other half was released into MMS-containing media without auxin treatment. At the indicated time points samples were taken for 2D gel, FACS and Western blot analysis. During quantification the highest value obtained for the X-molecules was considered as 100%. The efficiency of Hmo1 depletion was analyzed with anti-Hmo1 antibody via immunoblotting. Pgk1 was used for loading control.

The culmination of error-free DDT is the formation of SCJs, later resolved by Sgs1-Top3 (Branzei *et al*, 2008). To address if Hmo1 also affects the formation or the stability of SCJs generated during error-free DDT, we studied by 2D gel electrophoresis the profile of replication intermediates arising at an early, efficient origin of replication, *ARS305*, when yeast cells replicate in media containing MMS (Fig2B). Because in *sgs1*Δ cells the processing of the resulting recombination intermediates is impaired and SCJs forming during error-free DDT accumulate (Liberi *et al*, 2005; Branzei *et al*, 2008), we used this genetic background as a tool to address a possible role for Hmo1 in this process. Furthermore, since *hmo1*Δ strains are slow-growing (Lu *et al*, 1996) and the profile of replication intermediates can be severely impacted by the cell-cycle/replication status, we used again the *hmo1-AID* degron system described above (see Fig1D) to induce Hmo1 depletion. *sgs1*Δ *hmo1-AID* cells grow normally, but Hmo1 depletion at the beginning of replication correlated with a decrease in the amount of SCJs (Fig2B, 60–120 min panels), which gradually increased following prolonged MMS treatment (Fig2B, 180–240 min panels). Thus, Hmo1 facilitates SCJ formation/stability in the same time window with the one reported for Rad5-Mms2-Ubc13 (Karras *et al*, 2013), being predominant early during replication. Furthermore, these results indicate that Hmo1 depletion does not significantly impair the functionality of the salvage recombination pathway that normally promotes SCJ formation later in the cell-cycle (Branzei *et al*, 2008; Karras *et al*, 2013).

To examine if the above 2D gel results might reflect a role for Hmo1 in promoting SCJ stability rather than their formation, we used again an *sgs1Δ hmo1-AID* strain but induced Hmo1-AID depletion after the initiation of SCJ formation (1 h after the cells were released from G1 arrest into S phase, Supplementary Fig S2). Although under these conditions Hmo1 depletion also occurred efficiently, it did not anymore correlate with reduced SCJ levels (Supplementary Fig S2), in contrast to its effect at the beginning of replication (Fig2B, 60–120 min panels). Thus, following genotoxic stress, Hmo1 facilitates the usage of the Rad5 pathway, promoting template switching accompanied by SCJ formation early in S phase.

### Hmo1 is a novel regulator of the DDT pathway choice that acts in parallel with Elg1 and Srs2

**Figure 3:**
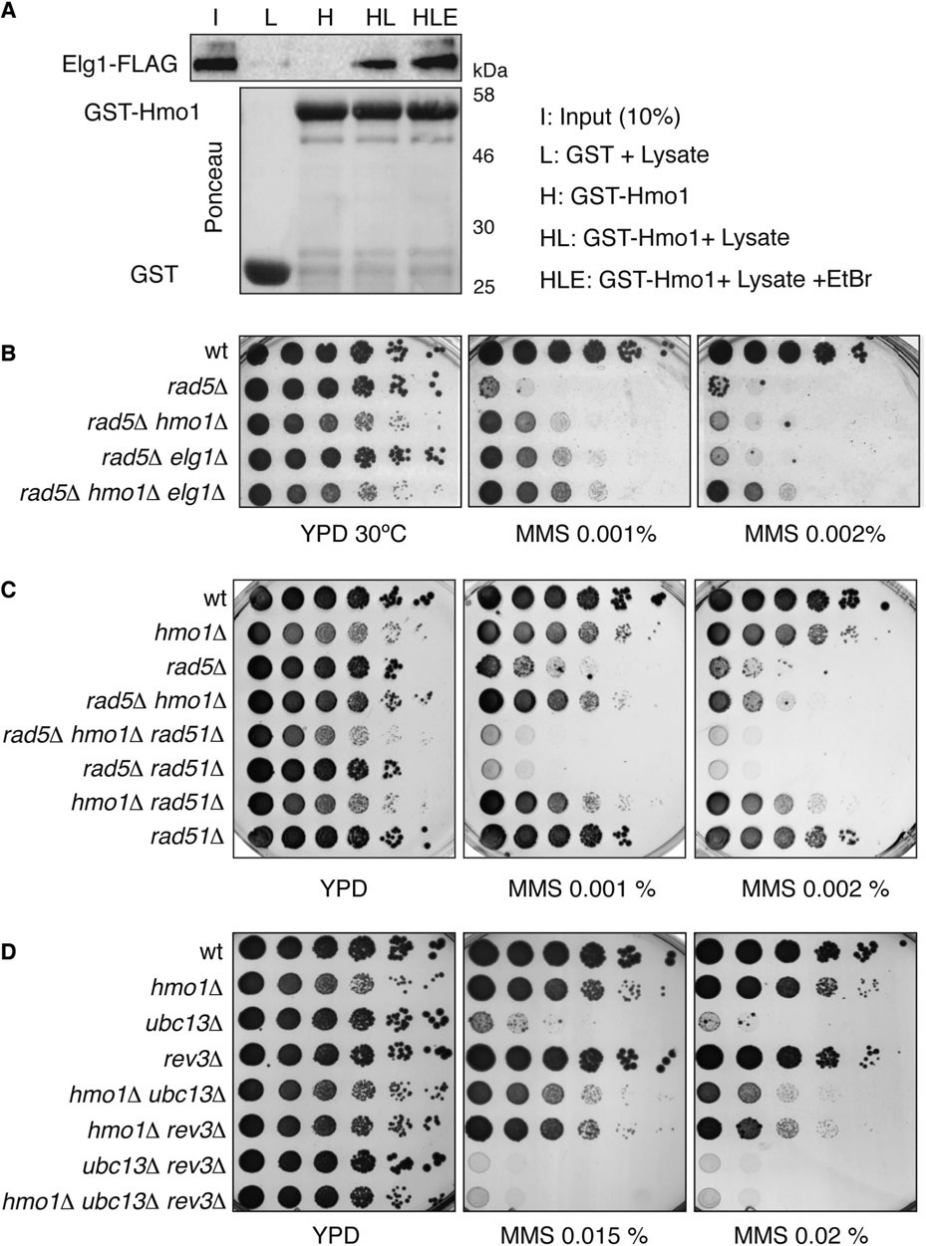
Hmo1 acts in parallel with Elg1 and Srs2 to promote Rad5-mediated error-free DDT

To understand the molecular mechanism by which Hmo1 facilitates the execution of the Rad5 pathway, we attempted to identify Hmo1 interacting proteins, using a candidate approach as well as yeast two-hybrid screens. We found initially by two-hybrid that Elg1, a regulator of the Rad5 pathway and a binding partner of PCNA (Parnas *et al*, 2010; Kubota *et al*, 2013), interacts physically with Hmo1. We then examined this interaction by *in vivo* pull-down assays. To this end, we purified recombinant GST and GST-Hmo1*,* immobilized these proteins on glutathione-sepharose beads, and incubated the beads with total cell lysates prepared from Elg1-FLAG yeast strains. In this way, we found that Elg1 is efficiently pulled-down to Hmo1 beads, even when the extract was treated with ethidium bromide, thus suggesting that the interaction between Hmo1 and Elg1 is not bridged by DNA (Fig3A).

Hmo1 interacts physically with Elg1. *In vivo* pull-down assay. Recombinant GST-Hmo1 protein was tested for its ability to bind endogenous Elg1. The amount of GST and GST-Hmo1 protein used is shown by Ponceau staining. Total cell lysates prepared from yeast cells expressing Elg1-FLAG tagged strain (HY1976) were incubated with GST or GST-Hmo1 in the presence or absence of ethidium bromide. The protein complex formed on the beads was analyzed by immunoblotting using anti-FLAG antibody.

*HMO1* and *ELG1* deletions additively rescue the MMS sensitivity of *rad5Δ*. wt (HY4104), *rad5Δ* (HY4098), *rad5Δ hmo1Δ* (HY4127), *rad5Δ elg1Δ* (HY4056) and *rad5Δ hmo1Δ elg1Δ* (HY4073) cells were spotted.

*HMO1* deletion rescues the MMS sensitivity of *rad5Δ* cells by suppressing the recombination pathway. wt (FY0113), *hmo1Δ* (HY3957), *rad5Δ* (HY0516), *rad5Δ hmo1Δ* (HY1518), *rad5Δ hmo1Δ rad51Δ* (HY3943), *rad5Δ rad51Δ* (HY3948), *hmo1Δ rad51Δ* (HY3946) and *rad51Δ* (HY2651) strains were spotted.

The survival of *hmo1Δ ubc13Δ* cells in MMS depends on the mutagenic pathway involving the translesion synthesis polymerase Rev3. wt (FY0090), *hmo1Δ* (HY1508), *ubc13Δ* (FY1490), *rev3Δ* (HY4416), *hmo1Δ ubc13Δ* (HY3960), *hmo1Δ rev3Δ* (HY4439), *ubc13Δ rev3Δ* (HY4417) and *ubc13Δ rev3Δ hmo1Δ* (HY4440) strains were spotted.

The *elg1Δ* mutation suppresses the sensitivity of *rad5Δ*, *ubc13Δ*, and *mms2Δ* cells to MMS by a similar degree as the one conferred by *hmo1Δ* (Fig3B, note the growth defect associated with *hmo1Δ*). However, the combination of *hmo1Δ* and *elg1Δ* mutations leads to a much better suppression of the *rad5Δ* sensitivity than the one conferred by single mutations (Fig3B), attesting to the individual roles of Elg1 and Hmo1 in error-free DDT regulation and indicating that the distinct modulatory actions of Elg1 and Hmo1 on the Rad5 pathway are potentially coordinated via their physical interaction.

While the mechanism by which Elg1 regulates the Rad5 pathway remains elusive, it possibly involves a joint action of Elg1 with Srs2, the other known regulator of the Rad5-mediated DDT branch that acts by affecting the choice of the recombinational repair pathway (Rong *et al*, 1991; Papouli *et al*, 2005; Pfander *et al*, 2005). The interplay between Srs2 and Elg1 in error-free DDT regulation was suggested by their preferential binding to SUMOylated PCNA (Papouli *et al*, 2005; Pfander *et al*, 2005; Parnas *et al*, 2010) and the observation that simultaneous deletion of *SRS2* and *ELG1* leads to a growth impairment that is partly improved by a SUMOylation-defective allele of PCNA (Parnas *et al*, 2010). The proposed mechanism envisages that while Srs2 disrupts toxic recombination events and makes space for the action of the Rad5 pathway (Aboussekhra *et al*, 1992; Krejci *et al*, 2003; Veaute *et al*, 2003; Papouli *et al*, 2005; Pfander *et al*, 2005), Elg1 may help unload (SUMOylated) PCNA from chromatin to facilitate DNA repair (Parnas *et al*, 2010; Kubota *et al*, 2013).

To further investigate the mechanism by which Hmo1 modulates Rad5-mediated DDT, we aimed at identifying the DDT pathways required for viability in *rad5Δ hmo1Δ* and *ubc13Δ hmo1Δ* cells. Similarly to the case previously elucidated for Srs2 (Rong *et al*, 1991; Aboussekhra *et al*, 1992; Papouli *et al*, 2005; Pfander *et al*, 2005), we found that the viability of *rad5Δ hmo1Δ* depended on the salvage recombination pathway involving Rad51 (Fig3C) and the recently identified 9-1-1 activities (Karras *et al*, 2013) (Supplementary Fig S3A), but not on Ubc13 (Supplementary Fig S3B). In addition, Hmo1 was not required for the viability of *rad5Δ srs2Δ* cells exposed to MMS (Supplementary Fig S3C, note the growth defect associated with *hmo1Δ*). This latter result, together with the 2D gel analysis data showing that Hmo1 is dispensable for the formation of late SCJs (Fig2B), likely arising via the action of the salvage pathway of recombination (Branzei *et al*, 2008; Karras *et al*, 2013), indicates that Hmo1 is not required for the execution of the salvage recombination pathway. Furthermore, we found that the viability conferred by *HMO1* deletion in mutants defective in the PCNA polyubiquitylation pathway of template switching depends on the TLS polymerase, Rev3 (Fig3D). Thus, defects in the PCNA polyubiquitylation pathway in WT cells causes MMS hypersensitivity, whereas additional inhibition of *HMO1* cells allows other recombination- and TLS-mediated DDT pathways to operate efficiently. Together, these results allow us to conclude that Hmo1 is a new regulator of the error-free DDT pathway, acting in parallel with Srs2 and Elg1, to facilitate the Rad5-mediated error-free DDT pathway and influencing DDT pathway choice.

### Uncoupling error-free DDT pathway choice from SCJ formation during template switching

**Figure 4:**
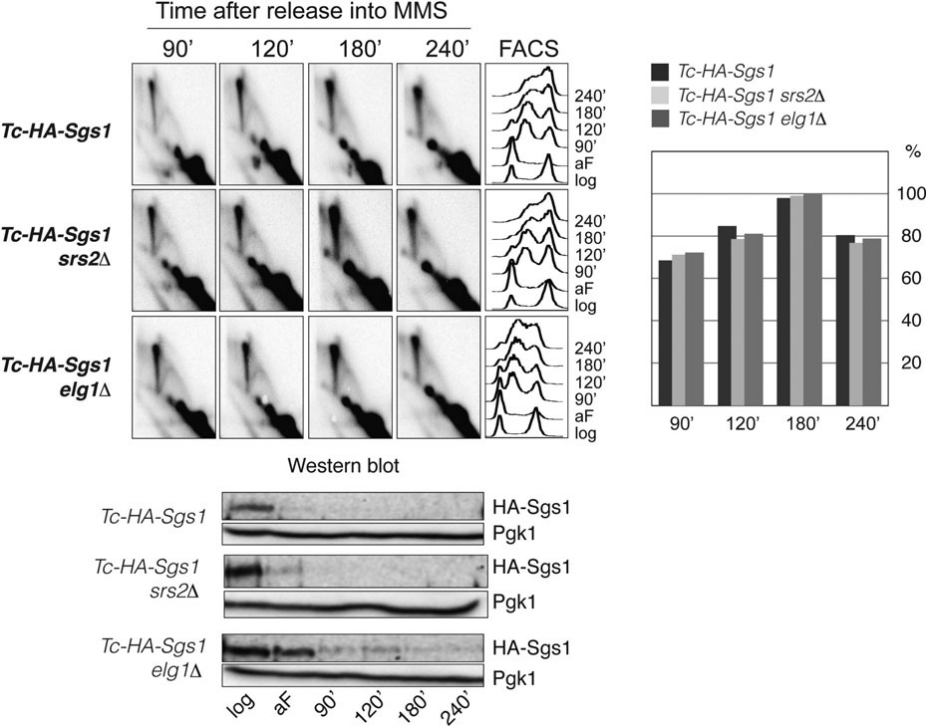
Srs2 and Elg1 involved in DDT pathway choice regulation are not required for SCJ formation during template switching *Tc-Sgs1* (HY4017), *Tc-Sgs1 elg1Δ* (HY4320) and *Tc-Sgs1 srs2Δ* (HY4352) cells were synchronized with alpha-factor (aF) in the presence of tetracycline and released into YPD media containing 0.033% MMS in the presence of tetracycline. At the indicated time points samples were taken for 2D gel, FACS and Western blot analysis. During quantification, the highest value obtained for the X-molecules accumulating was considered as 100%. Depletion of Sgs1 (tagged with 3HA) was followed by Western blot using anti-HA antibody. Pgk1 was used for loading control.

The functionality of the Rad5 error-free DDT is reflected in the ability of cells to timely fill in DNA gaps (Torres-Ramos *et al*, 2002; Zhang & Lawrence, 2005) with the transient formation of SCJ intermediates (Branzei *et al*, 2008; Minca & Kowalski, 2010; Karras *et al*, 2013). However, whether the Rad5 pathway regulators, which direct lesions into the Rad5 pathway and/or facilitate its usage, also impact on SCJ formation is not known. The individual mutation of *srs2Δ* in a WT background does not affect SCJ levels (Liberi *et al*, 2005), and we found a similar profile of replication intermediates in WT and *elg1Δ* cells (Supplementary Fig S4). However, the low levels of SCJ intermediates and their transient nature in WT cells do not allow for conclusive answers in what regards a possible role for Srs2 and Elg1 in SCJ formation. In an *sgs1Δ* background, in which SCJ persistence facilitates the identification of genetic requirements (Liberi *et al*, 2005; Branzei *et al*, 2008; Vanoli *et al*, 2010), deletion of *SRS2* or *ELG1* leads to synthetic lethality or a slow growth phenotype (Mullen *et al*, 2001; Parnas *et al*, 2010), incompatible with the correct assessment of replication intermediate status by 2D gel analysis. To address a possible role for Srs2 and Elg1 in SCJ generation, we established a conditional mutant for *SGS1* (*Tc-SGS1*) in which Sgs1 translation is prevented upon addition of tetracycline (Kotter *et al*, 2009). Using this conditional allele, we could deplete Sgs1 and allow SCJ accumulation during replication (Fig4 and data not shown). Deletion of *SRS2* and *ELG1* in *Tc-SGS1* strains did not affect cell fitness, thus making them suitable for 2D gel analysis of replication intermediates arising in one cell cycle. When Tc-Sgs1 depletion was induced during replication, *srs2Δ* and *elg1Δ* mutations did not discernibly reduce SCJ accumulation (Fig4). These results reveal that the previously identified regulators of the Rad5 pathway usage, Elg1 and Srs2, which suppress *rad5Δ* sensitivity to MMS, do not affect SCJ formation during template switching. Thus, the function of guiding DDT pathway choice is uncoupled from the one(s) required for SCJ formation, and Hmo1 participates in both of these processes.

### Hmo1-mediated DNA bending facilitates error-free DDT by template switching

**Figure 5:**
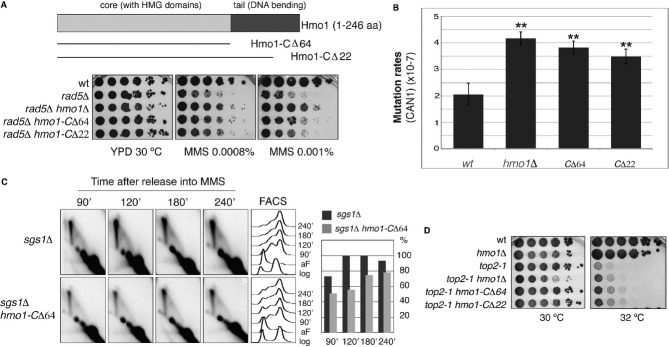
The C-terminal tail of Hmo1 required for DNA bending promotes Rad5-mediated error-free DDT and the formation of sister chromatid intertwinings *in vivo*

We next aimed at addressing if changes in DNA topology induced by Hmo1-mediated DNA bending underlie its roles in DDT pathway choice or SCJ formation. Similar to mammalian HMGB proteins, Hmo1 contains two DNA-binding domains termed box A and box B, and a lysine rich C-terminal tail (Fig5A). Of the DNA-binding domains of Hmo1, only box B corresponds to a consensus HMG box, while box A shows weak similarity. The HMG box is typically about 80 amino acids long and adopts an L-shaped fold composed of three α-helices. DNA binding, which occurs from the minor groove through intercalation of one or two hydrophobic residues, results in a sharp DNA bend and helical underwinding (Weir *et al*, 1993; Hardman *et al*, 1995). Biochemical studies indicated that box B is crucial for DNA binding, while box A plays only minor roles, affecting DNA bending by its interaction with the C-terminal tail of Hmo1 (Kamau *et al*, 2004; Bauerle *et al*, 2006; Xiao *et al*, 2010). The role of box A in bending is not fully understood as for certain assays measuring DNA bending, box A is dispensable (Xiao *et al*, 2010). In contrast, it has been clearly noted that the C-terminal tail of Hmo1 is crucial for DNA bending: Hmo1 C-terminal truncation variants are defective in DNA bending, while their DNA-binding affinity *per se* is not diminished (Bauerle *et al*, 2006; Xiao *et al*, 2010).

Scheme of Hmo1 and C-terminal truncation (Hmo1-CΔ) alleles. The Hmo1 C-terminal tail deletion partly suppresses the hypersensitivity of *rad5Δ* to MMS. wt (HY4104), *rad5Δ* (HY4098), *rad5Δ hmo1Δ* (HY4127), *rad5Δ hmo1-CΔ64* (HY4091) and *rad5Δ hmo1-CΔ22* (HY4108) strains were spotted.

Hmo1 prevents spontaneous mutagenesis and its C-terminal tail is required for this function. Spontaneous mutagenesis at the *CAN1* locus is shown for the indicated mutants. wt (FY0108), *hmo1Δ* (HY1508), *hmo1-CΔ64* (HY3893) and *hmo1-CΔ22* (HY3895) cells were used. Values and associated error-bars represent averages and their standard deviations from 3 independent experiments. ** denotes a highly significant *P*-value (*P* < 0.01).

Hmo1 promotes SCJ formation during template switching via its C-terminal tail. *sgs1Δ* (FY1060) and *sgs1Δ hmo1-CΔ64* (HY4303) cells were synchronized with alpha-factor (aF) and released into YPD media containing 0.033% MMS. At the indicated time points samples were taken for 2D gel and FACS analysis. In the quantification, the highest value obtained for the X-molecules accumulating was considered as 100%.

The C-terminal tail of Hmo1 is deleterious in *top2-1* mutants. wt (FY1296), *hmo1Δ* (HY1507), *top2-1* (HY3362), *top2-1 hmo1Δ* (HY3363), *top2-1 hmo1-C*Δ*64* (HY3890) and *top2-1 hmo1-CΔ22* (HY3892) were spotted at permissive (30 °C) and semi-permissive (32 °C) temperatures for *top2-1*.

To study the effect of Hmo1-mediated DNA bending in DDT regulation, we deleted the C-terminal tail of Hmo1 to construct *hmo1-CΔ22* and *hmo1-CΔ64* mutants (Fig5A and Supplementary Fig S5A). These Hmo1 variants are stable and *hmo1-CΔ22/64* strains do not show the growth defects characteristic of *hmo1Δ* (Fig5A), suggesting that they are proficient in certain Hmo1 functions as also suggested by their previous biochemical characterization (Bauerle *et al*, 2006; Xiao *et al*, 2010). In what regards DDT pathway choice, we found that both *hmo1-CΔ*22 and *hmo1-CΔ64* alleles resembled *hmo1Δ* in their ability to suppress the *rad5Δ* sensitivity to MMS, although their effect was smaller than that of *hmo1Δ* (Fig5A, DF5 background). We note that in a different yeast background, W303, in which the suppression conferred by *hmo1Δ* to *rad5Δ* is weaker than the one observed in DF5, the *hmo1-CΔ64* mutation suppresses *rad5*Δ sensitivity to MMS to the same degree as *hmo1*Δ (Supplementary Fig S5B). The reason underlying these background differences is unclear to us. Nevertheless, considering that *hmo1-CΔ* mutations partly suppress *rad5Δ* sensitivity to MMS in two different yeast backgrounds, we conclude that the C-terminus of Hmo1 is at least partly involved in DDT pathway choice. In addition, the *hmo1*-*CΔ* alleles showed increased spontaneous mutation rates (Fig5B) and impaired damage-bypass via SCJ formation (Fig5C), similarly to *hmo1*Δ or Hmo1 depletion (Figs5B and 2B), respectively, thus substantiating the important role of the C-terminal tail of Hmo1 in error-free DDT. In all, these results suggest that Hmo1-mediated DNA bending facilitates channeling of DNA lesions into the Rad5 error-free DDT pathway and the execution of template switching via SCJ formation.

### Hmo1-mediated DNA bending facilitates formation of sister chromatid intertwinings

To further examine if Hmo1 role in template switching and SCJ formation (Figs2B and 5C) is related to its role in altering DNA topologies in a manner that might facilitate sister chromatid interactions, we purified recombinant Hmo1 full-length, as well as an Hmo1 variant with a truncated C-terminus, and incubated increasing amounts of these Hmo1 proteins with Top1-relaxed plasmids. Addition of Hmo1, but not of the C-terminal truncated Hmo1 variant, promoted a gel retardation of the relaxed topoisomers (Supplementary Fig S5C). Since the migration pattern of topoisomers following Hmo1 addition is the one expected for supercoiled and nicked catenated plasmid dimers (Kegel *et al*, 2011), these findings indicate that Hmo1 mediates the formation or stabilization of catenanes/hemicatenanes via its C-terminal tail.

Hmo1 was previously reported to be deleterious in *top2* mutants for reasons that remained elusive. We asked if Hmo1 deleterious effect is related to its role in stabilizing catenanes/hemicatenanes via its C-terminal domain (Supplementary Fig S5C). Indeed, similarly to *hmo1Δ*, the *hmo1-CΔ22* and *hmo1-CΔ64* alleles also partially suppressed the temperature sensitivity phenotype of *top2-1* cells (Fig5D). Together, these results indicate that the DNA-bending activity of Hmo1 mediates the formation of sister chromatid intertwinings that, under conditions of replication stress, facilitate replication by template switching.

## Discussion

Replication is associated with DNA structural and topological changes as well as with specific post-translational modifications of DNA damage response factors that assist DDT and replication completion (Branzei & Foiani, 2010). RPA-coated ssDNA, accumulating following replication under conditions of genotoxic stress, activates the replication checkpoint (Mec1/Ddc2 in yeast and ATR/ATRIP in mammals), as well as DDT pathways (Zou & Elledge, 2003; Branzei & Foiani, 2010). The latter event appears to be mediated through RPA-dependent recruitment of Rad18 (Davies *et al*, 2008), which together with Rad6 and the Rad5-Mms2-Ubc13 complex promotes PCNA modification with mono- and polyubiquitin chains (Hoege *et al*, 2002), and induces translesion synthesis- and error-free-mediated DDT, respectively (Stelter & Ulrich, 2003; Papouli *et al*, 2005; Pfander *et al*, 2005; Branzei *et al*, 2008). The choice of the DDT pathway is crucial for genome integrity, as mutagenesis and hyper-recombination can lead to accumulation of deleterious mutations and chromosomal rearrangements that threaten genome integrity and promote cancer formation (Nik-Zainal *et al*, 2012; Alexandrov *et al*, 2013). In addition, while a correlation between replication timing and mutation rates was established (Lang & Murray, 2011), the genome surveillance mechanisms that promote genome integrity by facilitating error-free DDT early during replication remain largely unknown.

So far two well conserved mechanisms related to PCNA modifications have been shown to influence DDT pathway choice: one is related to the PCNA mono/poly-ubiquitylation status and affects the labor distribution between translesion synthesis-mediated error-prone damage bypass and Rad5-mediated error-free DDT, whereas the other regulatory mechanism is mediated by PCNA SUMOylation (Bergink & Jentsch, 2009; Branzei & Foiani, 2010). According to the current view, transient PCNA SUMOylation during S phase prevents unwanted recombination from occurring during replication. Factors such as Srs2 in yeast and PARI in human cells that directly bind to SUMOylated PCNA (Papouli *et al*, 2005; Pfander *et al*, 2005; Parnas *et al*, 2010; Moldovan *et al*, 2011), or Elg1/ATAD5 that interacts with SUMOylated PCNA in yeast (Parnas *et al*, 2010) and regulates the levels of PCNA (ubiquitylation) in human cells (Lee *et al*, 2010), affect genome stability likely by regulating the mechanism through which cells tolerate DNA lesions.

In addition to these protein interactions and post-translational modifications that affect DDT signaling and DDT pathway choice, replication is associated with various DNA topological changes. These topological transitions include accumulation of positive supercoil ahead of the replication forks, partly compensated by the rotation of the replisome along the DNA helix and accompanied by the formation of precatenanes behind replication forks (Postow *et al*, 2001; Wang, 2002), hemicatenations of the sister chromatids behind replication forks (Lucas & Hyrien, 2000; Lopes *et al*, 2003) and formation of sister chromatid bridges when replication forks pass through chromatin loops containing transcribed regions (Bermejo *et al*, 2009). HMGB proteins bind to hemicatenated/catenated structures *in vitro* (Bianchi *et al*, 1989) and Hmo1 may stabilize sister chromatid bridges proposed to arise at intergenic loci during replication (Bermejo *et al*, 2009). Moreover, HMGB proteins bind DNA with low sequence specificity, and their binding to DNA affects chromatin architecture by inducing sharp DNA bends and helical underwinding (Thomas & Travers, 2001; Stros, 2010). However, if and how chromatin architecture affects replication and the choice of the DNA repair pathway remained to date largely unknown.

Our present work revealed that the chromatin architectural HMGB protein, Hmo1, promotes the error-free DDT pathway during replication via at least two specific functions. First, Hmo1 facilitates channeling of replication-associated lesions towards the Rad5 pathway of error-free DDT, while preventing the salvage pathway of recombination (Fig3C) and mutagenic bypass (Figs3D and 5B), thus contributing to the temporal separation and usage of template switching early during replication (Lang & Murray, 2011). We envisage that Hmo1-mediated bending may synergize with Elg1-mediated transactions (see Fig3A and B) to fine-tune the levels of chromatin associated PCNA, setting the stage for error-free DNA repair (Fig2A) and limiting the replication errors forming during damage-bypass (Fig5B). Secondly, we found that Hmo1 facilitates template switching by promoting SCJ formation (Fig2B). These functions of Hmo1 are both coincident with early DNA replication and are mediated by its C-terminal domain (Figs2 and 5), which is crucial for Hmo1-mediated DNA bending and architectural/topological changes (Supplementary Fig S5C and Fig S5D). In all, these results suggest that topological changes associated with DNA replication facilitate error-free DDT by template switching, and thus impact on genome integrity.

**Figure 6:**
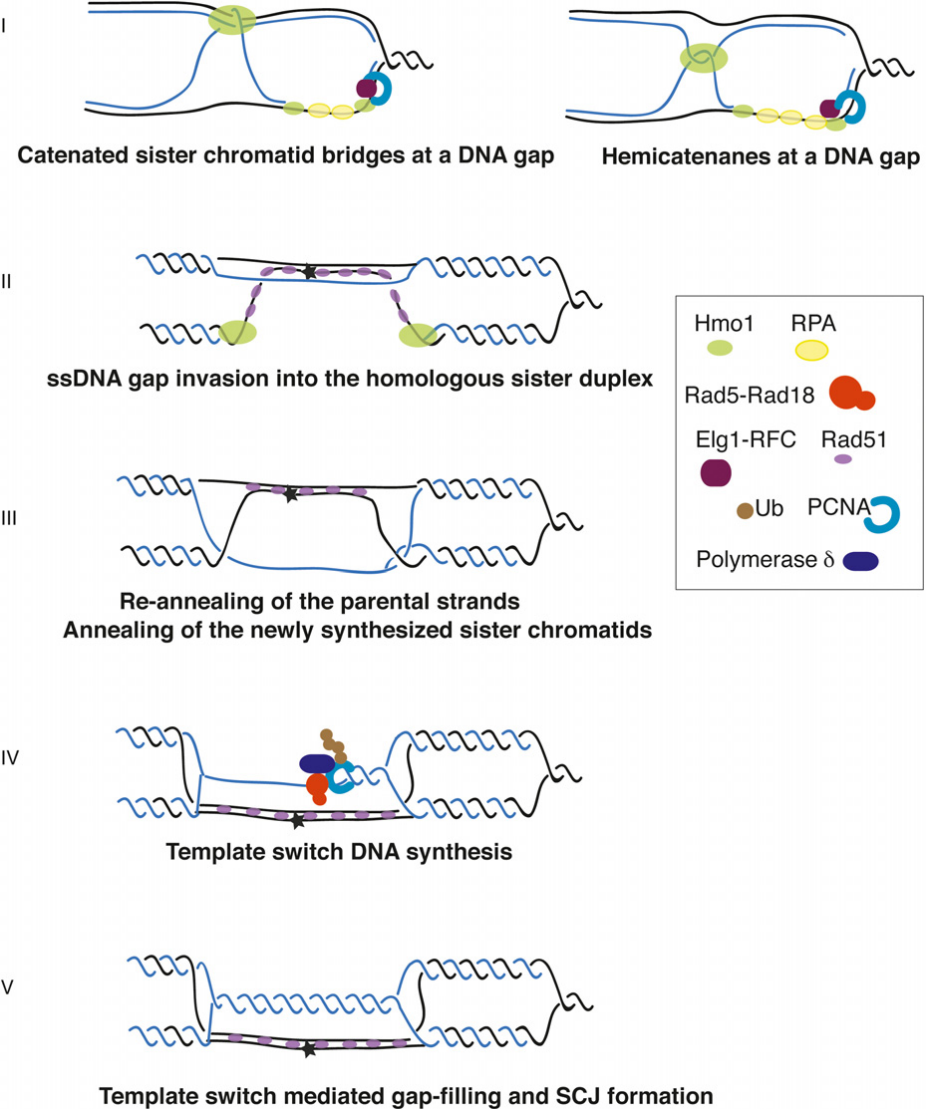
Hmo1 influences the S-phase chromosomal architecture creating a context favourable for error-free DDT by template switching A hypothetical model for Hmo1-mediated topological transitions promoting template switching. Parental DNA is shown in black, the newly synthesized DNA in blue. The asterisk indicates a DNA lesion. Sister chromatid bridges (Bermejo *et al*, 2009) and hemicatenane structures (Lopes *et al*, 2003) arising during replication could be stabilized by Hmo1 (Bianchi *et al*, 1989; Jaouen *et al*, 2005) (I). These topological constrains can facilitate gap-filling via template switching by bringing in proximity the homologous sister duplex (I). In addition to this, or alternatively, via its ability to bend DNA, Hmo1 may promote looping of a DNA region containing the DNA gap, facilitating strand invasion by inducing extensive pairing/annealing of the invading Rad51 filament with the homologous duplex (II). This would lead to re-annealing of the parental strands (in black), and exposure of the newly synthesized strand (in blue) (III). Extension of the 3' end proximal to the gap by Polδ and Rad5-mediated PCNA polyubiquitylation (Branzei *et al*, 2008; Vanoli *et al*, 2010) using the newly synthesized chromatid as template (IV) would lead to formation of template switch intermediates containing SCJs (V).

In addition to sister chromatid bridges proposed to form upon encountering of replication forks with transcription units (Bermejo *et al*, 2009), replication-dependent SCJs, hypothesized to represent hemicatenanes, may form behind replication forks even in unperturbed conditions (Lucas & Hyrien, 2000; Benard *et al*, 2001; Lopes *et al*, 2003; Robinson *et al*, 2007). When replication-related X-molecules, encounter GGA/TTC repeats, homology-driven junctions substitute the original asymmetric hemicatenanes (Follonier *et al*, 2013). Thus, hemicatenanes or related topological structures may facilitate homology-mediated annealing to the same template strand in case of direct repeats. By analogy, in case of replication in the presence of genotoxic stress, we speculate that topological constrains arising during replication may facilitate annealing of the gap-containing region to the homologous sister duplex and promote template switching (Fig6, I). As HMGB proteins show high affinity for cruciform structures (Bianchi *et al*, 1989; Stros *et al*, 2004; Jaouen *et al*, 2005), it is possible that Hmo1, via its ability to bind hemicatenanes and catenated sister chromatid bridges, prevents their dissolution upon encountering ssDNA gaps, thereby facilitating annealing of the ssDNA gap into the homologous duplex (Fig6, II) and formation of subsequent SCJs generated during template switching (Fig6, III–V).

In addition to the model proposed above, and not mutually exclusive, Hmo1 may promote template switching and SCJ formation via its reported ability to induce formation of chromatin loops via DNA-bending (Xiao *et al*, 2010). We envisage that under conditions of DNA damage, these chromatin loops will often contain ssDNA gap regions and would mediate homology search by engaging in inter-molecular interaction with the sister homologous duplex (Fig6, II). We note that a closed circular nucleofilament of the Rad51 bacterial ortholog, RecA, efficiently invades a duplex (Bianchi *et al*, 1983). Furthermore, the Rad51 nucleofilament contained in the loop would promote extensive pairing with the homologous sequence from the donor duplex, thereby facilitating homologous recombination (De Vlaminck *et al*, 2012). This would lead to efficient re-annealing of the parental strands (Mozlin *et al*, 2008; Vanoli *et al*, 2010) and exposure of the newly synthesized strand for DNA synthesis (Fig6, III), thus facilitating template switching (Fig6, IV–V).

In conclusion, our results suggest that replication-associated chromatin architectural changes act as a novel layer of regulation, besides the molecular switch mediated by PCNA ubiquitylation/SUMOylation, to control DDT pathway choice and to promote error-free replication under conditions of genotoxic stress. Our findings thus establish a link between replication-associated topological changes and DDT pathway choice, highlighting the role of chromatin architecture as an important modulator of genome integrity, by setting the stage for error-free replication and DNA repair.

## Materials and Methods

### Yeast strains

The strains used in this study are derivatives of DF5 or W303. The relevant genotypes are shown in Supplementary Table 1

### Growing conditions, cell cycle arrests and drug treatments

Unless otherwise indicated, strains were grown at 30°C in YPD medium, synchronized with 2 μg/ml α-factor and released in 0.033% MMS.

### Genomic DNA extraction, FACS analysis and 2D gel technique

Purification of DNA was performed by the CTAB procedure; FACS and 2D gel analysis of DNA intermediates were performed as previously described (Branzei *et al*, 2008). DNA samples were analyzed by 2D gel using probes against *ARS305* following NcoI or EcoRV-HindIII digestion. Quantification of X-shaped intermediates was done using IMAGEQUANT software, as previously described (Branzei *et al*, 2008) and as detailed in the Supplementary Data 1. Each experiment was independently performed at least twice and a representative experiment is shown.

### ChIP-on-chip

These procedures are derived from the ChIP-on-chip protocol previously described (Bermejo *et al*, 2009) and detailed in the Supplementary Information Anti-PK SV5-Pk1 antibody (AbD Setotec) and anti-BrdU antibodies (MI-11-3 from MBL) were employed. ChIP-on-chip experiments were independently performed at least twice and a representative experiment is shown. Evaluation of the significance of protein cluster distributions was performed as described in (Bermejo *et al*, 2009).

### Two-hybrid screens

Yeast two-hybrid screening was performed by Hybrigenics Services, S.A.S., Paris, France (http://www.hybrigenics-services.com). Further information is given in the Supplementary Data 1.

### Mutagenesis assays

Spontaneous mutation rates were estimated using the maximum-likelihood approach and as described in the Supplementary Data 1.

### Hmo1 protein expression and purification

The procedure used to express Hmo1 and Hmo1-CΔ64 proteins is detailed in the Supplementary Data 1.

### DNA supercoiling assay

The assay was performed by relaxing 1 μg of plasmid YIplac211 with 1 U of wheat-germ Topoisomerase I (Promega) for 1 h at 37°C. The indicated amounts (in μg) of full-length or C-terminal truncated Hmo1 were added and the reaction was left for the time indicated (15 or 60 min). The reactions were stopped by addition of 3% SDS and DNA ethanol-precipitated prior to resuspending and loading onto a 0.6% agarose gel in 1× TBE buffer. Electrophoresis was performed at 45 V for 15 h. We also performed phenolization prior to ethanol-precipitation, obtaining analogous results.

### *In vivo* pull-down assay

Approximately 5 μg of bacterially expressed GST and GST-Hmo1 proteins were immobilized on 30 μl of glutathione-Sepharose 4B beads. For *in vivo* pull-down assay, extracts were prepared from Elg1-FLAG cells arrested with α factor (G1) and released in YPD with and without 0.033% MMS for 20 min. Approximately 2.5 mg of total cell lysates were incubated with GST and GST-Hmo1 proteins at 4°C in Tris-HCl buffer (Tris pH 7.5, 150 mM NaCl, 1 mM DTT, 1 mM EDTA, 10% glycerol, 0.1% Triton X-100 and Protease inhibitor cocktail) for 2 h, in the presence or absence of 0.5 mg/ml of ethidium bromide. The beads were washed twice with Tris-HCl buffer and twice with Tris-HCl buffer containing 500 mM NaCl. The protein complexes formed on the beads were subjected to 10% SDS-PAGE and analysed by immunoblotting using anti-FLAG-M2 antibody (Sigma). The proteins were visualized by enhanced chemiluminescence (ECL), according to the manufacturer's instructions (Amersham ECL Plus).

## Supporting Information

Supplementary Data S1

Review Process File
