# Supplementary material for: DNA bending facilitates the error-free DNA damage tolerance pathway and upholds genome integrity
Source: EMBO J. 2014 Jan 31;33(4):327–40. doi: 10.1002/embj.201387425 (PMC3983681; doi:10.1002/embj.201387425)
Supplement: Supplementary file 1 [file embj0033-0327-sd1.pdf]

Supplementary Figures and Figure Legends

Figure S1 relates to Figure 1

Supplementary Figure 1

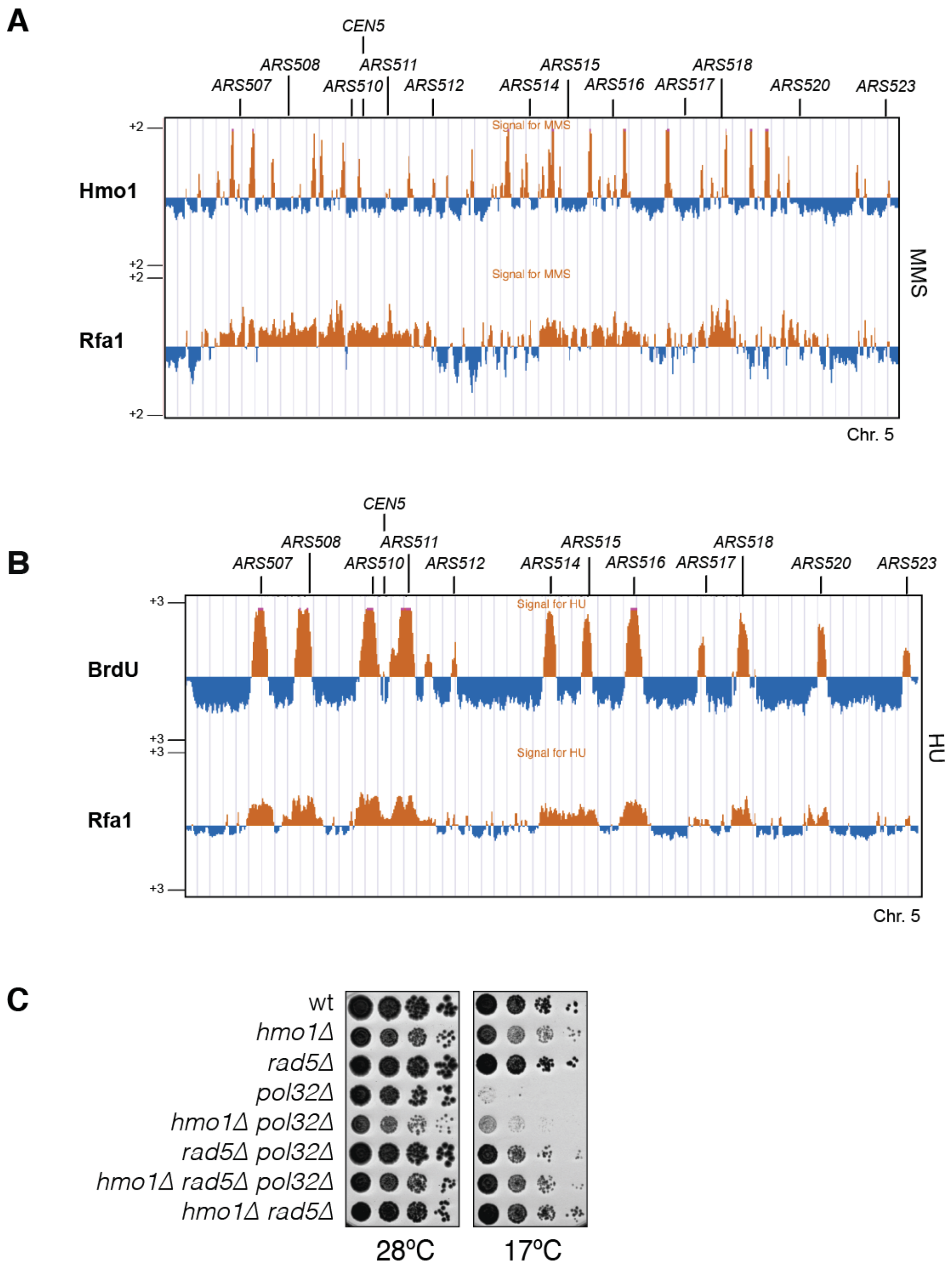

Figure S1. Genome-wide analysis of Hmo1 clusters following replication in the presence of genotoxic stress and characterization of *pol32* cold sensitivity suppression by *hmo1* . **(A)** Hmo1-6xPK (FY1687) and Rfa1-6xPK (HY3800) were released from G1 arrest in the presence of MMS 0.033% for 1 hour and processed for ChIP with antibodies specific to the PK epitope. Orange histogram bars in the y-axis show the average signal ratio of loci significantly enriched in the immunoprecipitated fraction along the indicated regions in log2 scale. Positions of *ARS* elements and *CENs* are indicated. **(B)** wt strains containing TK repeats to allow BrdU incorporation (FY1110) were released from G1 arrest in media containing 0.2M HU and BrdU for 90 min. Rfa1-6xPK (HY3800) strains were released in 0.2M HU media for 90 min. Samples were processed for ChIP with anti-BrdU antibodies or antibodies specific to the PK epitope. **(C)** wt (FY0090), *hmo1* (HY1508), *rad5* (Y1223), *pol32* (Y2593), *hmo1 pol32* (IP1136), *rad5 pol32* (Y2663), *hmo1 rad5 pol32* (IP1138), *hmo1 rad5* (IP1128) strains were spotted.
