# Supplementary material for: DNA bending facilitates the error-free DNA damage tolerance pathway and upholds genome integrity
Source: EMBO J. 2014 Jan 31;33(4):327–40. doi: 10.1002/embj.201387425 (PMC3983681; doi:10.1002/embj.201387425)
Supplement: Supplementary file 2 [file embj0033-0327-sd2.pdf]

Figure S2 relates to Figure 2

Supplementary Figure 2

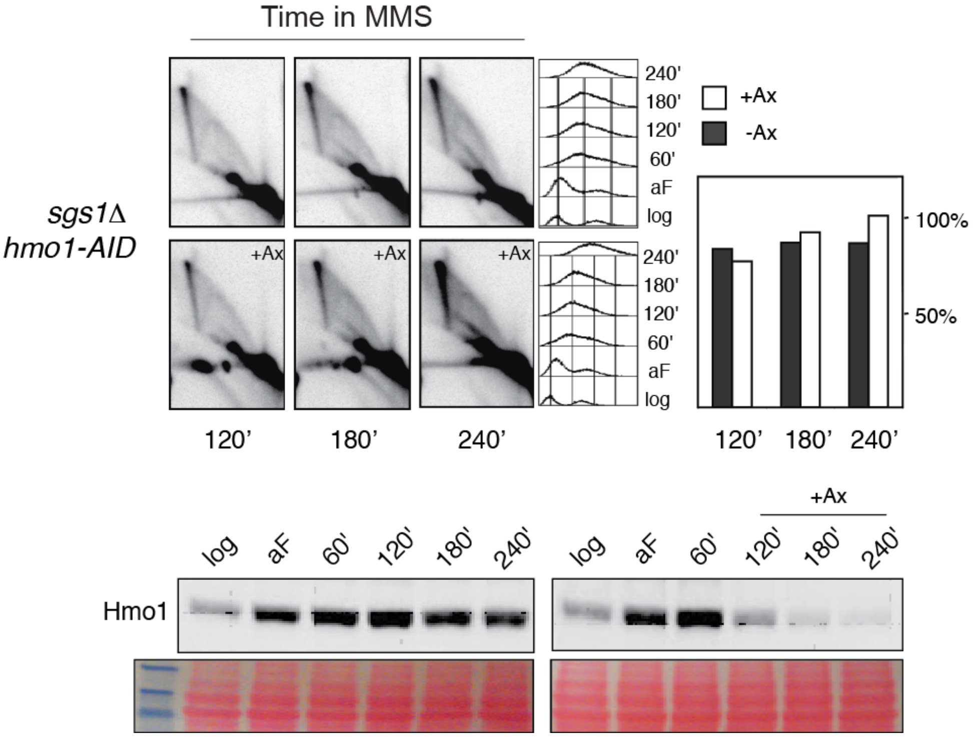

Figure S2. Hmo1 depletion does not interfere with the stability of SCJs formed during replication of damaged templates. 2D gel profile of replication intermediates of an *sgs1 hmo1-AID* conditional mutant (HY2176) following or not Hmo1 depletion by addition of auxin (Ax) 60 min after release from G1 arrest into YPD media containing 0.033% MMS. Also included are FACS profiles, X-molecule quantification and the western blot to control Hmo1 depletion, together with the Ponceau staining of samples as loading control. During quantification the highest value obtained for the X-molecules accumulating was considered as 100%.
