# Supplementary material for: DNA bending facilitates the error-free DNA damage tolerance pathway and upholds genome integrity
Source: EMBO J. 2014 Jan 31;33(4):327–40. doi: 10.1002/embj.201387425 (PMC3983681; doi:10.1002/embj.201387425)
Supplement: Supplementary file 3 [file embj0033-0327-sd3.pdf]

Figure S3 relates to Figure 3

Supplementary Figure 3

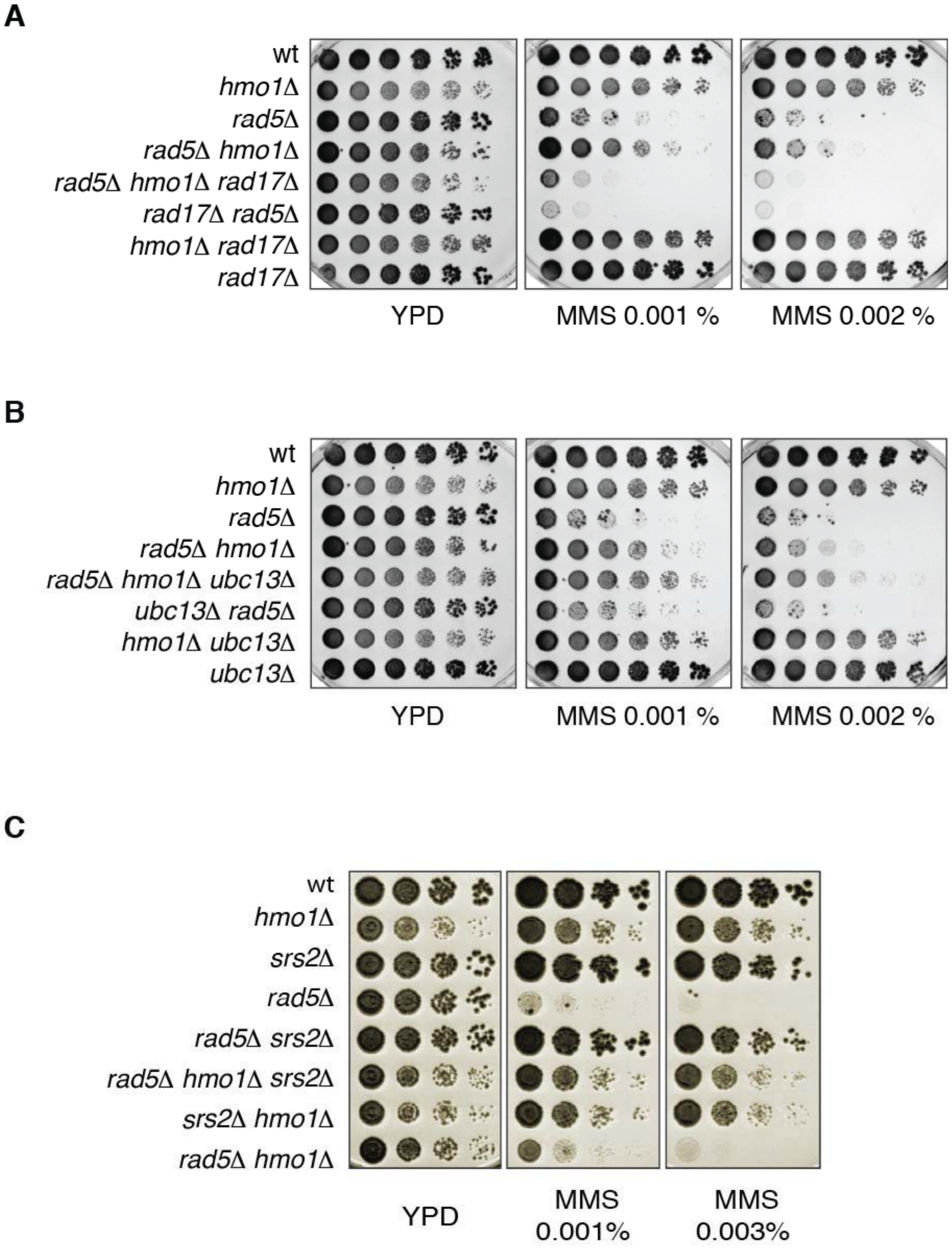

Figure S3. The viability of *rad5 hmo1* cells depends on the salvage recombination pathway, but not on Ubc13. **(A)** wt (FY0113), *hmo1* (HY3956), *rad5* (HY0516), *rad5 hmo1* (HY1518), *rad5 hmo1 rad17* (HY3939), *rad17 rad5* (HY3936), *hmo1 rad17* (HY3941), *rad17* (HY2224) strains were spotted. **(B)** wt (FY0113), *hmo1* (HY3957), *rad5* (HY0516), *rad5 hmo1* (HY1518), *rad5 hmo1 ubc13* (HY3944), *rad5 ubc13* (HY3958), *hmo1 ubc13* (HY3959), *ubc13* (FY1490) strains were spotted. **(C)** Hmo1 is not required for the salvage recombination pathway. Strains wt (FY0113), *hmo1* (HY1508), *srs2* (Y1572), *rad5* (Y1223), *rad5 srs2* (Y1576), *rad5 srs2 hmo1* (IP1126), *srs2 hmo1* (IP1130), *rad5 hmo1* (IP1128) strains were spotted.
