# Supplementary material for: DNA bending facilitates the error-free DNA damage tolerance pathway and upholds genome integrity
Source: EMBO J. 2014 Jan 31;33(4):327–40. doi: 10.1002/embj.201387425 (PMC3983681; doi:10.1002/embj.201387425)
Supplement: Supplementary file 4 [file embj0033-0327-sd4.pdf]

Figure S4 relates to Figure 4

Supplementary Figure 4

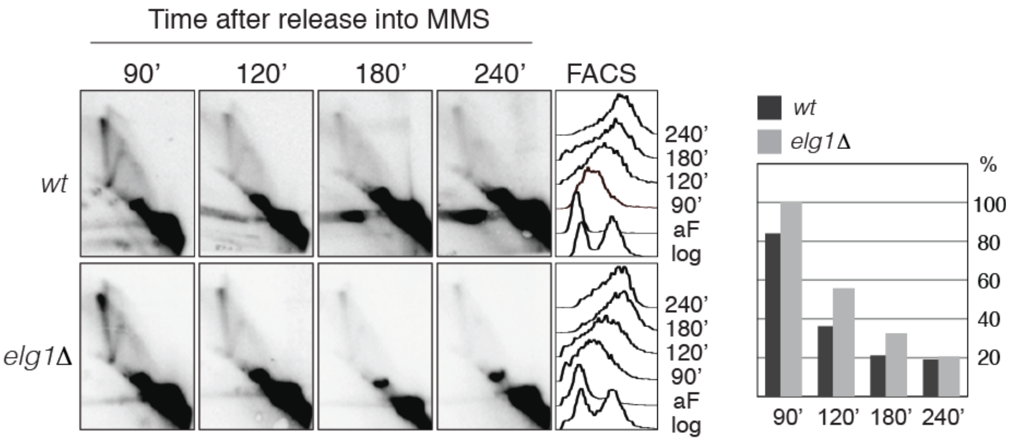

Figure S4. Null *ELG1* mutation does not reduce the amount of SCJs generated during template switching. wt (FY1000) and *elg1* (HY2003) cells were synchronized with alpha-factor (aF) and released into YPD media containing 0.033% MMS. At the indicated time-points samples were taken for 2D gel and FACS analysis. During quantification the highest value obtained for the X-molecules accumulating was considered as 100%.
