# Supplementary material for: DNA bending facilitates the error-free DNA damage tolerance pathway and upholds genome integrity
Source: EMBO J. 2014 Jan 31;33(4):327–40. doi: 10.1002/embj.201387425 (PMC3983681; doi:10.1002/embj.201387425)
Supplement: Supplementary file 5 [file embj0033-0327-sd5.pdf]

Figure S5 relates to Figure 5

Supplementary Figure 5

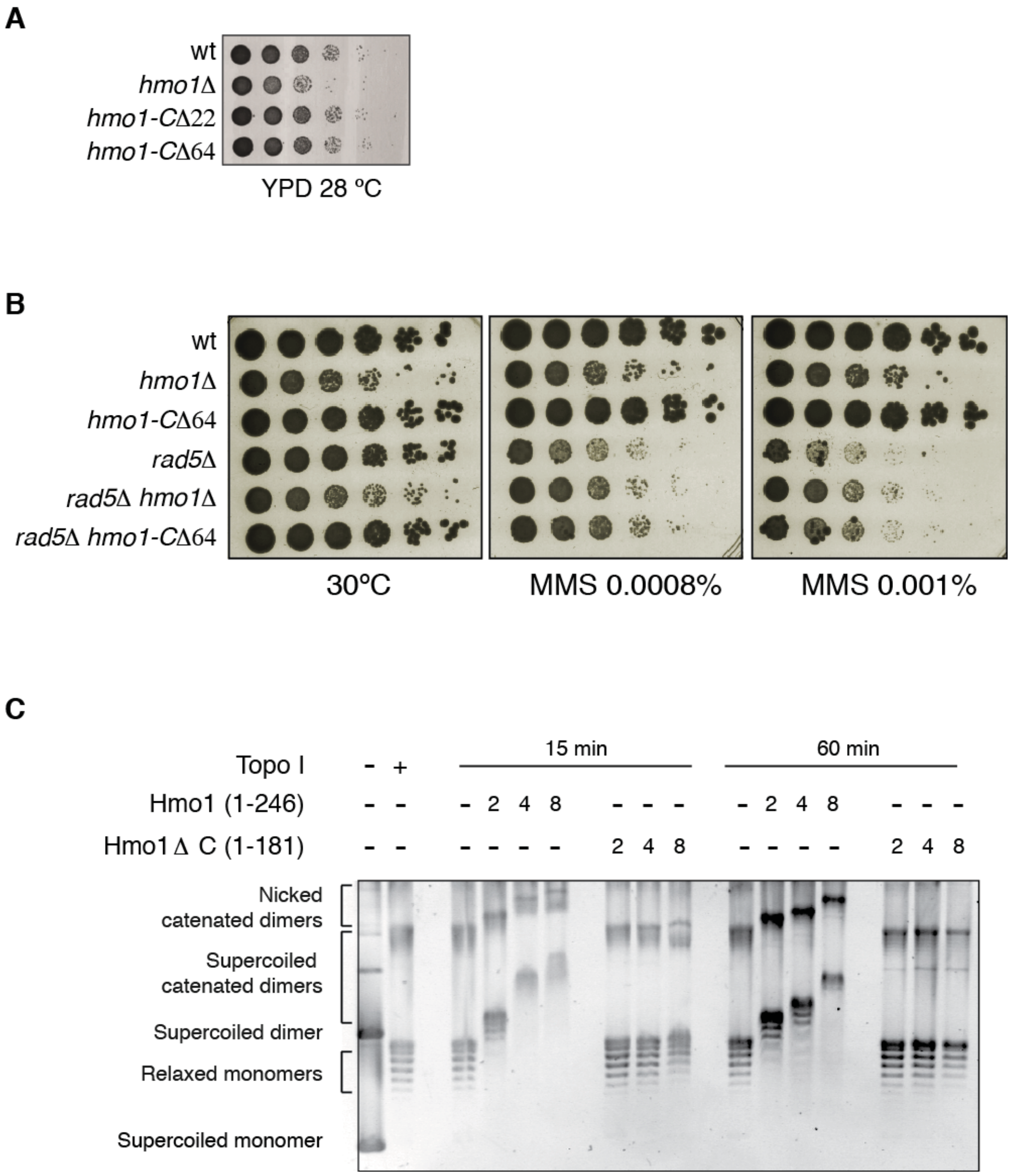

Figure S5. The C-terminal tail of Hmo1 is required for Hmo1-mediated Rad5 pathway activation in the presence of DNA damage and for promoting DNA catenation/hemicatenation *in vitro*. **(A)** Hmo1-C alleles show normal growth. wt (HY4103), *hmo1* (HY1508), *hmo1-C 22* (HY4113) and *hmo1-C 64* (HY4101) cells were spotted. **(B)** Hmo1 affects the Rad5 pathway via its C-terminal tail. wt (FY1296),

hmo1 (HY3662), *hmo1*-C 64 (HY3732), *rad5* (HY3664), *rad5 hmo1* (HY3632), rad5 *hmo1*-C 64 (HY3735) strains were spotted. **(C)** Hmo1 alters DNA topology in a C-terminal tail-dependent manner. Following relaxation of 1 g of YIplac211 plasmid for 1 hour with wheat-germ topoisomerase I (TopoI), increasing amounts (2, 4 or 8 g) of full-length or C-terminally truncated Hmo1 proteins were added and the reaction was allowed to proceed for 15 or 60 min. The topoisomers were separated in a 0.6% agarose gel.
