# Supplementary material for: DNA bending facilitates the error-free DNA damage tolerance pathway and upholds genome integrity
Source: EMBO J. 2014 Jan 31;33(4):327–40. doi: 10.1002/embj.201387425 (PMC3983681; doi:10.1002/embj.201387425)
Supplement: Supplementary file 6 [file embj0033-0327-sd6.pdf]

Supplemental Experimental Procedures

Supplementary Materials include 1 table, 5 figures, extended experimental procedures, and references.

Table S1. _Saccharomyces cerevisiae_ strains used in this study. The strains are listed in alphabetical/number ascending order order.

Strain	Genotype	Source
**FY0090**	***MATa*** *his3- 200 leu2-3, 112 lys2-801 trp1-1 (am) ura3-52* (DF5 background, wt)	Lab collection
**FY0108**	***MATa*** *his3- 200 leu2-3, 112 lys2-801 trp1-1 (am) ura3-52* (DF5 background, wt)	Lab collection
**FY0113**	***MAT*** *his3- 200 leu2-3, 112 lys2-801 trp1-1 (am) ura3-52* (DF5 background, wt)	Lab collection
**FY1000**	***MATa*** *ade2-1 can1-100 his3-11,-15 leu2-3,112 trp1-1 ura3-1 RAD5+* (W303 background, wt)	Lab collection
**FY1060**	**W303** Mata *sgs1 ::HisMX*	This study
**FY1110**	**W303** Mata *ura3::URA3/GPD-TK(7X)*	Lab collection
**FY1296**	***MATa*** *ade2-1 can1-100 his3-11,-15 leu2-3,112 trp1-1 ura3-1 RAD5+* (W303 background, wt)	Lab collection
**FY1487**	**W303** Mata but *arg4 BglII URA3::arg4 EcoRV::ura3-1 pol30-RR rad5-535*	Lab collection
**FY1490**	**DF5** Mata *ubc13 ::HPHNT1*	Lab collection
**FY1687**	**W303** Mata *Hmo1-6PK::HIS3*	Lab collection
**HY0516**	**DF5** Mat *rad5 ::HphMX4*	Lab collection
**HY0518**	**DF5** Mat *mms2 ::HPHMX4*	This study
**HY1507**	**W303** Mata *hmo1 ::KanMX6*	This study
**HY1508**	**DF5** Mat *hmo1 ::KanMX6*	This study
**HY1518**	**DF5** Mat *rad5 ::HphMX4 hmo1 ::His3MX4*	This study
**HY1519**	**DF5** Mat *mms2 ::HPHMX4 hmo1 ::His3MX6*	This study
**HY1976**	**W303** Mata *ELG1-10FLAG::KanMX4*	Lab collection
**HY2003**	**W303** Mata *elg1 ::KanMX*	Lab collection
**HY2174**	**W303** Mata *ura3-1::ADH1-OsTIR1-9 Myc (URA3) hmo1::hmo1-AID (KanMX4)*	This study
**HY2176**	**W303** Mata *ura3-1::ADH1-OsTIR1-9 Myc (URA3) hmo1::hmo1-aid(kanMX) sgs1 ::HPHMX4*	This study
**HY2224**	**DF5** Mata *rad17 ::HIS3*	Lab collection
**HY2651**	**DF5** Mata *rad51 ::loxP-KANMX-loxP*	Lab collection
**HY2682**	**W303** Mat *rad5 ::HPHMX4*	This study
**HY2706**	**DF5** Mata *pol32 ::klTRP1 hmo1 ::KanMX6*	This study
**HY2714**	**DF5** Mata *hmo1 ::KanMX6*	This study

**HY2719**	**DF5** Mata *pol32 ::klTRP1*	This study
**HY3362**	**W303** Mata *top2-1*	Lab collection
**HY3363**	**W303** Mata *top2-1 hmo1 ::His3MX4*	This study
**HY3632**	**W303** Mata *rad5 ::HPHMX4 hmo1 ::HIS3*	This study
**HY3633**	**W303** Mat *rad5 ::HPHMX4 hmo1 ::HisMX*	This study
**HY3662**	**W303** Mata *hmo1 ::KanMX6*	This study
**HY3664**	**W303** Mata *rad5 ::HPHMX4*	This study
**HY3732**	**W303** Mata *hmo1-C 64::KanMX4*	This study
**HY3735**	**W303** Mata *rad5 ::HPHMX4hmo1-C 64::KanMX4*	This study
**HY3800**	**W303** Mata *Rfa1-6PK::HIS3*	This study
**HY3890**	**W303** Mata *top2-1 hmo1-C 64::KanMX4*	This study
**HY3892**	**W303** Mata *top2-1 hmo1-C 22::KanMX4*	This study
**HY3893**	**DF5** Mata *hmo1-C 64::KanMX4*	This study
**HY3895**	**DF5** Mata *hmo1-C 22::KanMX4*	This study
**HY3936**	**DF5** Mata *rad5 ::NATN2 rad17 ::HIS3*	This study
**HY3939**	**DF5** Mata *rad5 ::NATN2 hmo1 ::KanMX4 rad17 ::HIS3*	This study
**HY3941**	**DF5** Mata *hmo1 ::KanMX4 rad17 ::HIS3*	This study
**HY3943**	**DF5** Mat *rad5 ::NATN2 hmo1 ::KanMX4 rad51 ::URA3*	This study
**HY3944**	**DF5** Mat *rad5 ::NATN2 hmo1 ::KanMX4 ubc13 ::HPHMX*	This study
**HY3946**	**DF5** Mat *hmo1 ::KanMX4 rad51 ::URA3*	Lab collection
**HY3948**	**DF5** Mat *rad5 ::NATN2 rad51 ::URA3*	Lab collection
**HY3956**	**DF5** Mata *hmo1 ::KanMX4*	This study
**HY3957**	**DF5** Mat *hmo1 ::KanMX4*	This study
**HY3958**	**DF5** Mata *rad5 ::NATN2 ubc13 ::HPHMX*	This study
**HY3959**	**DF5** Mat *hmo1 ::KanMX4 ubc13 ::HPHMX4*	This study
**HY3960**	**DF5** Mata *hmo1 ::KanMX4 ubc13 ::HPHMX4*	This study
**HY4017**	**W303** Mata *sgs1::pADH1-tc3-3xHA-Sgs1 (KanMX)*	Lab collection
**HY4056**	**DF5** Mata *rad5 ::NATN2, elg1 ::HIS3*	This study
**HY4073**	**DF5** Mata *rad5 ::NATN2, elg1 ::HIS3, hmo1 ::KanMX4*	This study
**HY4091**	**DF5** Mata *rad5 ::NATN2, hmo1-C 64::KanMX4*	This study
**HY4098**	**DF5** Mata *rad5 ::NATN2*	This study
**HY4101**	**DF5** Mat *hmo1-C 64::KanMX4*	This study
**HY4103**	**DF5** Mat	This study
**HY4104**	**DF5** Mata *his3-delta200 leu2-3,112 lys2-801 trp1-1 (am) ura3-52* (DF5 background, wt)	This study
**HY4108**	**DF5** Mata *rad5 ::NATN2 hmo1-C 22::KanMX4*	This study
**HY4113**	**DF5** Mat *hmo1-C 22::KanMX4*	This study
**HY4127**	**DF5** Mata *rad5 ::NATN2, hmo1 ::KanMX4*	This study
**HY4303**	**W303** Mata *sgs1 ::NATN2hmo1-C 64::KanMX4*	This study
**HY4320**	**W303** Mata *sgs1::pADH1-tc3-3xHA-Sgs1 (NATN2) elg1 ::KanMX4*	This study
**HY4324**	**W303** Mata *S-Hmo1::natNT2*	This study
**HY4325**	**W303** Mata *ura3-1::ADH1-OsTIR1-9 Myc (URA3) G2-*	This study

	Hmo1::natNT2	
**HY4352**	**W303** Mata *sgs1::pADH1-tc3-3xHA-Sgs1 (HPHMX4) srs2 ::KANMX*	This study
**HY4355**	**W303** Mata *S-Hmo1::natNT2 rad5 ::HPHMX4*	This study
**HY4359**	**W303** Mata *ura3-1::ADH1-OsTIR1-9 Myc (URA3) G2-Hmo1::natNT2 rad5 ::HPHMX4*	This study
**HY4416**	**DF5** Mata *rev3 ::HIS3*	This study
**HY4417**	**DF5** Mata *ubc13 ::HPHMX4 rev3 ::HIS3*	This study
**HY4439**	**DF5** Mat *hmo1 ::KanMX4 rev3 ::HIS3*	This study
**HY4440**	**DF5** Mata *ubc13 ::HPHMX4 hmo1 ::KanMX4 rev3 ::HIS3*	This study
**IP1126**	**DF5** Mat *rad5::KanMX4, srs2::klTRP1, hmo1 ::KanMX4*	This study
**IP1128**	**DF5** Mat *rad5::KanMX4, hmo1 ::KanMX4*	This study
**IP1130**	**DF5** Mat *srs2::klTRP1, hmo1 ::KanMX4*	This study
**IP1136**	**DF5** Mat *hmo1 ::KanMX6 pol32::klTRP1*	This study
**IP1138**	**DF5** Mat *hmo1 ::KanMX6 rad5::natNT2 pol32::klTRP1*	This study
**Y1223**	**DF5** Mat *rad5::KanMX4*	Lab collection
**Y1572**	**DF5** Mat *srs2::klTRP1*	Lab collection
**Y1576**	**DF5** Mat *rad5::KanMX4, srs2::klTRP1*	Lab collection
**Y1630**	**W303** Mata *siz1 ::His3MX6*	Lab collection
**Y2593**	**DF5** Mat *pol32::klTRP1*	Lab collection
**Y2620**	**DF5** Mata *ubc13 ::hphNT1*	Lab collection
**Y2663**	**DF5** Mata rad5::natNT2 *pol32::klTRP1*	Lab collection
