# Supplementary material for: DNA bending facilitates the error-free DNA damage tolerance pathway and upholds genome integrity
Source: EMBO J. 2014 Jan 31;33(4):327–40. doi: 10.1002/embj.201387425 (PMC3983681; doi:10.1002/embj.201387425)
Supplement: Supplementary file 7 [file embj0033-0327-sd7.pdf]

Extended experimental procedures

Yeast strains

The strains used in this study are derivatives of W303 or DF5 and were prepared by genetic crosses and standard disruption techniques (Longtine et al., 1998). They are listed in Table S1.

Hmo1 and Sgs1 conditional depletions

0.2 mg/ml auxin (Sigma-Aldrich) was added to *sgs1 hmo1-AID* mutant (HY2176) or to *hmo1-AID* cells (HY2174) cells either 40 min before effective G1 arrest or 60 min after release into YPD medium containing 0.033% MMS. When auxin was added before G1 arrest, cells were washed with auxin-containing YP and released into YPD media containing 0.2 mg/ml auxin. To deplete Sgs1 using *Tc-SGS1* alleles (*Tc-SGS1*, *TC-SGS1 elg1* and *Tc-SGS1 srs2*), tetracycline was added to G1 arrested cultures at a final concentration of 0.6 mM.

Quantification of X-shaped intermediate signals in 2D-gels

Quantification was performed using the IMAGEQUANT software. For each time point, areas corresponding to the monomer spot (M), the X-spike signal and a region without any replication intermediates as background reference were selected and the signal intensities (SI) in percentage of each signal were obtained. The values for the X and monomer were corrected by subtracting from the SI value the background value after the latter was multiplied for the ratio between the dimension of the area for the intermediate of interest and for background. Thus, the values for X and M were calculated in the following way:

Value for X = SI (Xs) - [SI (background) (area (Xs)/area (background)];

Value for M= SI (M) - [SI (background) (area (M)/area (background)].

The relative signal intensity for the X was then determined by dividing the value for X with the sum of the total signals (the sum of the X and monomer values). The resulting values for X signals were then normalized and converted to percentage by using the highest value number of X for each experiment as 100 and normalizing the other values to it.

Two-hybrid screens

Yeast two-hybrid screening was performed by Hybrigenics Services, S.A.S., Paris, France (http://www.hybrigenics-services.com). The coding sequence for aminoacids 1-220 of *S. cerevisiae HMO1* was PCR-amplified and cloned into pB27 as a C-terminal fusion to LexA (N-LexA-Hmo1-C) and into pB66 as a C-terminal fusion to Gal4 DNA-binding domain (N-Gal4-Hmo1-C). The constructs were checked by sequencing and used as a bait to screen a genomic *S.cerevisiae* library constructed into pP6. pB27, pB66 and pP6 derive from the original pBTM116 (Vojtek & Hollenberg, 1995), pAS2ΔΔ and pGADGH plasmids, respectively (Fromont-Racine et al, 1997). For the LexA bait construct, 33 million clones (6-fold the complexity of the library) were screened using a mating approach with HGX13 (Y187 *ade2-101::loxP-kanMX-loxP, MAT□)* and L40□Gal4 (*MATa*) yeast strains as previously described (Fromont-Racine et al, 1997). A total of 353 His+ colonies were selected on a medium lacking tryptophan, leucine and histidine. For the Gal4 construct, 22 million clones (4-fold the complexity of the library) were screened using the same mating approach with Y187 (*MAT□*) and CG1945 (*MATa*) yeast strains. A total of 7 His+ colonies were selected on a medium lacking tryptophan, leucine and histidine. The prey fragments of the positive clones were amplified by PCR and sequenced at their 5' and 3' junctions. The resulting sequences were used to identify the corresponding interacting proteins in the GenBank database (NCBI) using a fully automated procedure. A confidence score (PBS, for Predicted Biological Score) was then attributed to each interaction as previously described (Formstecher et al, 2005).

ChIP-on-chip

The ChIP-on-chip protocol previously described (Bermejo et al, 2009). The microarray data were submitted online using GEO linking under the series number GSE46260 (http://www.ncbi.nlm.nih.gov/geo/query/acc.cgi?token=hlcdzegwygiqepy&acc=GSE46260). For ChIP-on-chip experiments, we crosslinked cells with 1% formaldehyde (30 min at room temperature for Hmo1-PK, 30 minutes at room temperature and overnight at 4°C for Rfa1-PK).

Hmo1 protein expression and purification

We generated a hexahistidine-SUMO tagged Hmo1 full length (1-246 aa) and truncation constructs containing the Hmo1 N-terminus (residues 1-181) in SUMO tagged vector, as well as Hmo1 full length and C-terminus truncated constructs (residues 1-181) in GST-tagged expression vector (pGEX-6P-1). Both GST- and hexahistidine-SUMO tagged constructs were expressed in *Escherichia coli* BL21 DE3-Codon Plus-RIL (Novagen). Cells were grown at 37ºC till OD_{660} reached 0.5-0.6, and then temperature was decreased to 20ºC and expression was induced with 0.4 mM of isopropyl-1-thio-D-galactopyranoside. The culture was continued for 12 hrs, following which cells were harvested and resuspended in lysis buffer (25 mM Tris-HCl, pH 7.6, 800 mM NaCl, 10 mM imidazole, 5% glycerol). The cells were lysed by sonication for 10 minutes, and then the lysate was clarified by centrifugation at 40,000g for 1 hr.

GST-tagged Hmo1 proteins used for pull-downs were purified on a glutathione-sepharose column (GSTrap HP, GE healthcare). After elution with a 20 mM of L-glutathione containing buffer, the fusion proteins were dialyzed against a buffer containing 25 mM Tris-HCl, pH 7.6, 100 mM NaCl and 2.5% glycerol at 4ºC. GST-tagged, Hmo1 full length and C-terminus truncated, proteins were concentrated to 10 and 15 mg mL^{-1} respectively at 4 ºC in Amicon Ultra-15 mL (Millipore) 10 KDa cut-off concentrator. The hexahistidine-SUMO fusion protein was purified on a nickel-charged column (HisTrap HP, GE healthcare). After elution with 500 mM imidazole containing buffer, the fusion protein was cleaved with Ulp1 protease (Invitrogen Life Technologies) at 15 U ml^{-1} during 16 hr dialysis against buffer containing 25 mM Tris-HCl, pH 7.6, 150 mM NaCl and 5% glycerol at 4ºC. The protein was further purified by cation-exchange (HiTrap Heparin HP) and gel filtration chromatography (HiLoad Superdex 200 26/60) equilibrated with 15 mM Tris-HCl, pH 7.6, 500 mM NaCl and 2% Glycerol. Purified Hmo1 full-length and C-terminal truncated Hmo1 proteins were concentrated to 20, and 15 mg mL^{-1} respectively at 4 ºC using the Amicon Ultra-15 mL (Millipore) 10 KDa cut-off concentrator.

Antibodies

We used polyclonal Hmo1 antibodies (S. Brill lab) for Hmo1 Western Blot (Lu et al, 1996). Polyclonal antibodies against PCNA are described (Hoege et al., 2002), and antibodies against 3-phosphoglycerate kinase (Pgk1) were from Invitrogen (459250). Antibodies used for ChIP-on-chip experiments were the V5-tag antibody (anti-PK) from AbD Serotec (MCA1360) and the anti-BrdU MI-11-3 from MBL.

Mutagenesis Assays

Spontaneous mutagenesis at the *CAN1* locus was assessed by measuring the canavanine resistant fraction of parallel, saturated populations. 10 individual YPD cultures were setup with a 1:20000 inoculum from an overnight culture that should contain the smallest number of inactivating mutations at the *CAN1* locus possible. Cultures were incubated with constant shaking at 30 C for 36 h in order to promote the acquisition of spontaneous mutations and appropriate dilutions were washed with sterile water and were plated on YPD plates or SC plates lacking arginine, but containing canavanine (80 g/ml) (SC-Arg+CAN). Spontaneous mutation rates were estimated using the maximum-likelihood approach.

References

Bermejo R, Capra T, Gonzalez-Huici V, Fachinetti D, Cocito A, Natoli G, Katou Y, Mori H, Kurokawa K, Shirahige K, Foiani M (2009) Genome-organizing factors Top2 and Hmo1 prevent chromosome fragility at sites of S phase transcription. *Cell* **138:** 870-884

Formstecher E, Aresta S, Collura V, Hamburger A, Meil A, Trehin A, Reverdy C, Betin V, Maire S, Brun C, Jacq B, Arpin M, Bellaiche Y, Bellusci S, Benaroch P, Bornens M, Chanet R, Chavrier P, Delattre O, Doye V, Fehon R, Faye G, Galli T, Girault JA, Goud B, de Gunzburg J, Johannes L, Junier MP, Mirouse V, Mukherjee A, Papadopoulo D, Perez F, Plessis A, Rosse C, Saule S, Stoppa-Lyonnet D, Vincent A, White M, Legrain P, Wojcik J, Camonis J, Daviet L (2005) Protein interaction mapping: a Drosophila case study. *Genome research* **15:** 376-384

Fromont-Racine M, Rain JC, Legrain P (1997) Toward a functional analysis of the yeast genome through exhaustive two-hybrid screens. *Nature genetics* **16:** 277-282

Lu J, Kobayashi R, Brill SJ (1996) Characterization of a high mobility group 1/2 homolog in yeast. *J Biol Chem* **271:** 33678-33685

Vojtek AB, Hollenberg SM (1995) Ras-Raf interaction: two-hybrid analysis. *Methods in enzymology* **255:** 331-342
