# Supplementary material for: DNA bending facilitates the error-free DNA damage tolerance pathway and upholds genome integrity
Source: EMBO J. 2014 Jan 31;33(4):327–40. doi: 10.1002/embj.201387425 (PMC3983681; doi:10.1002/embj.201387425)
Supplement: Supplementary file 8 [file embj0033-0327-sd8.pdf]

THE
EMBO
JOURNAL

Manuscript EMBO-2013-87425

DNA bending facilitates the error-free DNA damage tolerance pathway and upholds genome integrity

Victor Gonzalez-Huici, Barnabas Szakal, Madhusoodanan Urulangodi, Ivan Psakhye, Federica Castellucci, Demis Menolfi, Eerappa Rajakumara, Marco Fumasoni, Rodrigo Bermejo, Stefan Jentsch, and Dana Branzei

Corresponding author: Dana Branzei, IFOM FIRC Institute of Molecular Oncology

Review timeline:

Submission date:	15 November 2013
Editorial Decision:	25 November 2013
Revision received:	01 December 2013
Accepted:	03 December 2013

Editor: Hartmut Vodermaier

Transaction Report:

(Note: With the exception of the correction of typographical or spelling errors that could be a source of ambiguity, letters and reports are not edited. The original formatting of letters and referee reports may not be reflected in this compilation.)

Please note that the manuscript was previously reviewed at another journal and the reports were taken into account in the decision making process at The EMBO Journal. Since the original reviews are not subject to EMBO's transparent review process policy, the reports and author response cannot be published.

1st Editorial Decision 25 November 2013

Thank you again for transferring your manuscript together with the previous comments and responses to The EMBO Journal for our consideration. After having read and assessed all this material, I further sent it to an expert in the field and trusted advisor of the journal, for arbitrating input and feedback on the overall significance and suitability of this work for The EMBO Journal. Based on these considerations and the positive advisory comments copied below, I am pleased to inform you that we would happy to consider the study further for publication in our journal.

I would therefore like to invite you to submit, using the link provided below, a final version incorporating the following editorial points:

- please consider modifying certain parts of the manuscript (abstract, intro, discussion, and possibly title) to better highlight the important conceptual aspect of chromatin architecture as key modulator of genome surveillance, as emphasized in your rebuttal letter and as stressed by our arbitrator's comments (see below).

- please amend the manuscript with a brief 'Author Contribution' description, next to the Acknowledgement and Conflict of Interest statements

- in order to make the primary data more accessible and more directly represented, we encourage the inclusion of figure source data for gels, blots and autoradiographs, especially in cases where not a full gel but just a crop is shown in either main or supplementary figure panels. This should be in the

form of a single PDF/JPG/GIF file per figure comprising the original, uncropped and unprocessed blot scans/photographs, labelled with the appropriate figure/panel number and molecular weight markers; further annotation would clearly be useful but is not essential. These files can be uploaded upon resubmission selecting "Figure Source Data" as object type, and they would be linked as such to the respective figures in the online publication of your article.

I hope you will be able to return your this ultimate revision to us as early as possible, should you have any questions in this regard please don't hesitate to let me know. I look forward to receiving your final version!

_____

REFEREE REPORTS:

Referee #1:

Arbitrating referee 1 - comments:

I have now carefully read the manuscript from Gonzalez-Huici et al and studied all the correspondence from the previous submission. This is arguably a complicated matter but after considering all 'pros and cons' I do have a clear opinion, and I recommend publication. My main resins for this are as follows:

DNA transactions associated with DNA damage tolerance are inherently complex and these types of papers are in most cases truly appreciated only by a relatively small group of 'aficionados' (hence, most of them ultimately find their home in rather specialized journals). However, there is, in my view, an important aspect to this manuscript that actually highlights a general, and hitherto under-appreciated aspect of genome surveillance: the concept that nuclear architecture is an important determinant of genome maintenance and that proteins involved in topological and other biophysical aspects of DNA and chromatin metabolism can have important regulatory role (by 'setting the stage' for more dedicate repair reactions). Defects in such regulation might appear 'subtle' in short-term experiments, but turn cut crucial in maintaining healthy genomes throughout successive cell divisions. I think that the authors provide a compelling case for such role of DNA bending (mediated by Hmo1) in limiting errors during DNA damage bypass. I can see that they went a long way to improve also the textual part of the manuscript to convey the message to as broad audience as possible, and also their reasoning in the rebuttal is sound, in my opinion, especially during the second round.

In summary, I do agree with the authors that the involvement of genome architectural regulators such as Hmo1 in DNA damage tolerance is novel, and also that the relatively modest impact of Hmo1 manipulations in some assays is indeed what one would expect from such regulation. I am happy to recommend publication and I believe that by properly highlighting the role of chromatin architecture as an important modulator of genome surveillance, this paper can have a generally positive and inspiring impact in the field.

1st Revision - authors' response	01 December 2013

Point by point response to the editors' and referees' comments

I have now carefully read the manuscript from Gonzalez-Huici et al and studied all the correspondence from the previous submission. This is arguably a complicated matter but after considering all 'pros and cons' I do have a clear opinion, and I recommend publication. My main resins for this are as follows:

DNA transactions associated with DNA damage tolerance are inherently complex and these types of

© European Molecular Biology Organization

papers are in most cases truly appreciated only by a relatively small group of 'aficionados' (hence, most of them ultimately find their home in rather specialized journals). However, there is, in my view, an important aspect to this manuscript that actually highlights a general, and hitherto under-appreciated aspect of genome surveillance: the concept that nuclear architecture is an important determinant of genome maintenance and that proteins involved in topological and other biophysical aspects of DNA and chromatin metabolism can have important regulatory role (by 'setting the stage' for more dedicate repair reactions). Defects in such regulation might appear 'subtle' in short-term experiments, but turn cut crucial in maintaining healthy genomes throughout successive cell divisions. I think that the authors provide a compelling case for such role of DNA bending (mediated by Hmo1) in limiting errors during DNA damage bypass. I can see that they went a long way to improve also the textual part of the manuscript to convey the message to as broad audience as possible, and also their reasoning in the rebuttal is sound, in my opinion, especially during the second round.

In summary, I do agree with the authors that the involvement of genome architectural regulators such as Hmo1 in DNA damage tolerance is novel, and also that the relatively modest impact of Hmo1 manipulations in some assays is indeed what one would expect from such regulation. I am happy to recommend publication and I believe that by properly highlighting the role of chromatin architecture as an important modulator of genome surveillance, this paper can have a generally positive and inspiring impact in the field.

Response
We are very happy that the reviewer finds our work novel and important for understanding the role of chromatin architectural changes in DNA repair and genome integrity. Following also the editorís suggestions, we made textual modification in the manuscript (title, abstract, introduction, results and discussion) to highlight the important conceptual aspects of our work. We paid particular attention in highlighting how chromatin architectural changes act as a key and novel modulator to facilitate error-free DNA repair, thus upholding genome integrity. We also added a new result in Figure 3D (moving the original Figure 3D panel as S3C), which shows that the viability of ubc13 hmo1 cells depends on the translesion synthesis polymerase, Rev3, in line with the increased mutation rates observed in hmo1 mutant cells (Figure 5B). This result enforces the notion that Hmo1 facilitates the usage of the error-free DDT pathway; in the absence of Hmo1-mediated regulation, error-prone mechanisms promote damage-bypass at the detriment of genome stability. We thank the reviewer for the useful suggestions on how to pinpoint the general implications of our work.

© European Molecular Biology Organization
